# Risk of Falls and Fractures in Individuals With Cataract, Age-Related Macular Degeneration, or Glaucoma

**DOI:** 10.1001/jamaophthalmol.2023.5858

**Published:** 2023-12-28

**Authors:** Jung Yin Tsang, Alison Wright, Matthew J. Carr, Christine Dickinson, Robert A. Harper, Evangelos Kontopantelis, Tjeerd Van Staa, Luke Munford, Thomas Blakeman, Darren M. Ashcroft

**Affiliations:** 1 NIHR School for Primary Care Research, Centre for Primary Care and Health Services Research, University of Manchester, Manchester, United Kingdom; 2 NIHR Greater Manchester Patient Safety Research Collaboration, University of Manchester, Manchester, United Kingdom; 3 Division of Pharmacy and Optometry, School of Health Sciences, University of Manchester, Manchester, United Kingdom; 4 Manchester Royal Eye Hospital and Manchester Academic Health Sciences Centre, Manchester University NHS Foundation Trust, Manchester, United Kingdom; 5 Division of Informatics, Imaging and Data Sciences, School of Health Sciences, University of Manchester, Manchester, United Kingdom; 6 Health Organisation, Policy and Economics, School of Health Sciences, University of Manchester, Manchester, United Kingdom; 7 NIHR Applied Research Collaboration Greater Manchester, University of Manchester, Manchester, United Kingdom

## Abstract

**Importance:**

Three leading disease causes of age-related visual loss are cataract, age-related macular degeneration (AMD), and glaucoma. Although all 3 eye diseases have been implicated with falls and fracture risk, evidence is mixed, with the contribution of different eye diseases being uncertain.

**Objective:**

To examine whether people with cataract, AMD, or glaucoma have higher risks of falls or fractures than those without.

**Design, Setting, and Participants:**

This cohort study was a population-based study in England using routinely collected electronic health records from the Clinical Practice Research Datalink (CPRD) GOLD and Aurum primary care databases with linked hospitalization and mortality records from 2007 to 2020. Participants were people with cataract, AMD, or glaucoma matched to comparators (1:5) by age, sex, and general practice. Data were analyzed from May 2021 to June 2023.

**Exposures:**

For each eye disease, we estimated the risk of falls or fractures using separate multivariable Cox proportional hazards regression models.

**Main Outcomes:**

Two primary outcomes were incident falls and incident fractures derived from general practice, hospital, and mortality records. Secondary outcomes were incident fractures of specific body sites.

**Results:**

A total of 410 476 people with cataract, 75 622 with AMD, and 90 177 with glaucoma were matched (1:5) to 2 034 194 (no cataract), 375 548 (no AMD), and 448 179 (no glaucoma) comparators. The mean (SD) age was 73.8 (11.0) years, 79.4 (9.4) years, and 69.8 (13.1) years for participants with cataract, AMD, or glaucoma, respectively. Compared with comparators, there was an increased risk of falls in those with cataract (adjusted hazard ratio [HR], 1.36; 95% CI, 1.35-1.38), AMD (HR, 1.25; 95% CI, 1.23-1.27), and glaucoma (HR, 1.38; 95% CI, 1.35-1.41). Likewise for fractures, there were increased risks in all eye diseases, with an HR of 1.28 (95% CI, 1.27-1.30) in the cataract cohort, an HR of 1.18 (95% CI, 1.15-1.21) for AMD, and an HR of 1.31 (95% CI, 1.27-1.35) for glaucoma. Site-specific fracture analyses revealed increases in almost all body sites (including hip, spine, forearm, skull or facial bones, pelvis, ribs or sternum, and lower leg fractures) compared with matched comparators.

**Conclusions and Relevance:**

The results of this study support recognition that people with 1 or more of these eye diseases are at increased risk of both falls and fractures. They may benefit from improved advice, access, and referrals to falls prevention services.

## Key Points

**Question:**

Do people with cataract, age-related macular degeneration (AMD), or glaucoma have higher risks of falls or fractures?

**Findings:**

In this cohort study including 3 434 196 adults, we found an increased risk of falls in those with cataract, AMD, and glaucoma. For fractures, there was also an increased risk for those with cataract, AMD, and glaucoma.

**Meaning:**

The results of this study support recognition that people with 1 or more of these eye diseases are at increased risk of falls or fractures.

## Teaser

This cohort study examines data from general practice, hospital, and mortality records to determine whether individuals with cataract, age-related macular degeneration, or glaucoma have higher risks of falls or fractures than those without.

## Introduction

Three leading disease causes of age-related visual loss are cataract, age-related macular degeneration (AMD), and glaucoma, affecting more than 500 million people worldwide.^1^ Visual loss increases morbidity and mortality, including physical injuries, disability, poor cognition, and decreased mental health, leading to a reduction in activities of daily living and a loss of independence.^2,3^ The majority of these eye diseases are preventable or treatable, which may in turn reduce the risk of falls and related injuries, carrying important resource implications for global health and individual health systems dealing with an aging society.^2,4,5^ Falls are a major global health concern, particularly as the second leading cause of unintentional deaths due to injury worldwide.^6^ Annually, there are more than 650 000 deaths due to falls and more than 170 million falls resulting in short-term or long-term disability.^5^ This translates to an estimated cost of $23.3 billion annually in the United States and $1.6 billion in the United Kingdom.^7^

Poor vision is one of many risk factors for falls, but links to specific eye diseases remain inadequately defined. Visual function is vital for avoiding falls, with even relatively mild impairments in visual information affecting balance, posture, and gait.^2,8^ Yet in early stages of eye disease, patients are often asymptomatic and unaware of visual impairment.^9^ Both cataract and AMD mostly start affecting a single eye with a gradual onset in visual loss.^8^ In glaucoma, there is often insidious peripheral visual field loss, but the brain perceptually compensates for the missing areas by artificially completing the visual field.^10^ Although all 3 eye diseases have been implicated with falls and fracture risk, evidence is mixed, with current findings mainly derived from cross-sectional observations and having limited adjustment for established risk factors contributing to fall and fracture risk.^11,12,13,14^ Though smaller studies have reported an increased risk of falls and fractures, both the magnitude and contribution of each individual eye disease to these risks remain uncertain.^8,15,16,17,18^ Therefore, this study sought to determine the association of 3 leading age-related eye diseases with falls and fractures, adjusting for influential risk factors. The overarching aim was to investigate whether individuals with cataract, AMD, or glaucoma are at higher relative risk of falls or fractures compared with individuals without these eye diseases.

## Methods

### Study Design and Data Sources

This study was a population-based retrospective cohort study using the Clinical Practice Research Datalink (CPRD) GOLD and Aurum UK primary care databases.^19,20^ These contain anonymized longitudinal medical records from 2 of the most widely used clinical information systems in the United Kingdom, named Vision (GOLD) and EMIS Web (Aurum). The study was approved by CPRD’s independent scientific advisory committee and the Medicines and Healthcare products Regulatory Agency independent scientific advisory committee. Given the retrospective use of anonymized data, no informed consent was required. This study was conducted according to the guidelines of the Declaration of Helsinki, and the reporting followed the Strengthening the Reporting of Observational Studies in Epidemiology (STROBE) guideline.

### Setting

In the United Kingdom, the National Health Service is free at the point of care, with primary care functioning as gatekeepers of access to specialty care. Electronic records are adopted across all primary care practices, and data are collected daily from voluntarily enrolled practices by CPRD, which is then integrated with existing records and subjected to multiple quality checks.^21,22^ The data are nationally representative in terms of age, sex, and race and ethnicity, collated from more than 2200 primary care practices and including 18 million active patients across the United Kingdom, approximately 25% of the UK population.^19,20,23^ The patient-level data include detailed information on demographics, clinical events, prescriptions, and specialist referrals.

CPRD GOLD and Aurum data sets were combined for analyses as per previous studies.^23,24^ As there was a small overlap of practices that migrated clinical information software over time, a bridging file was used to drop practices who migrated from GOLD to Aurum to avoid double counting. Patient records were only included if deemed of acceptable quality for research (via a CPRD quality metric in GOLD). All included records were derived from practices based in England and linked at the patient level to Hospital Episode Statistics for hospitalization data, to the Office for National Statistics for mortality data, and by small area to the Index of Multiple Deprivation (IMD) 2015 stratified as quintiles.

### Participants and Cohort Delineation

**Figure 1.**
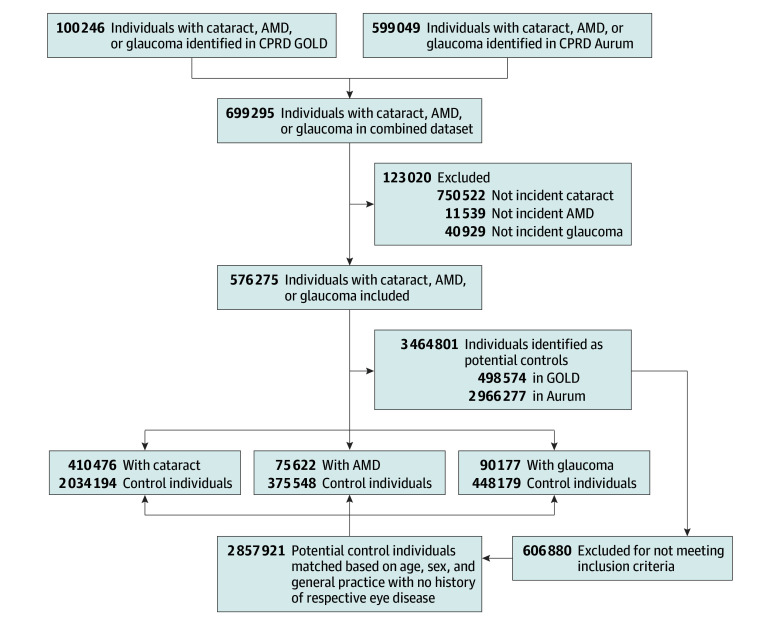
Study Flowchart Showing Cohorts Identified From the UK Clinical Practice Research Datalink (CPRD) GOLD and Aurum AMD indicates age-related macular degeneration.

Our study population included 3 separate cohorts (Figure 1) of adults 18 years and older, with cases defined as having a recorded diagnosis of cataract, AMD, or glaucoma (allowing concurrent eye disease within each cohort). These were identified from each database using Read codes (and additional SNOMED Clinical Terms EMIS-specific codes for CPRD Aurum) between April 1, 2007, and March 31, 2020, with previous studies demonstrating high validity in eye disease coding (eTable 1 in Supplement 1 contains lists of codes).^25,26^ However, we were unable to ascertain whether the eye disease was a monocular or binocular diagnosis.

Each case was matched with up to 5 corresponding comparators on age, sex, and general practice using incidence density sampling (99% cases were successfully matched with 5 comparators), with no recorded diagnosis of primary eye disease (ie, comparators were allowed to have a past diagnosis of the 2 other eye diseases). Study entry was defined as the first recorded eye-disease diagnosis date (at any point within the follow-up period but with at least 1-year registration within a general practice), with the end of follow-up defined as the earliest of death date, study end date (March 31, 2020), date of deregistration from a practice, or last data collection by the practice.

### Outcomes and Covariates

The 2 primary outcomes were rates of incident falls and incident fractures. These were identified using a predefined list of Read codes from primary care and *International Statistical Classification of Diseases and Related Health Problems, Tenth Revision,* codes from linked secondary care records.^27^ Diagnostic codes for pathological fractures were excluded to focus on trauma-related fractures. A secondary analysis examining the rate of site-specific fractures was also performed. This included all body sites of coded fractures with classifications guided by the National Institute for Health and Care Excellence guidelines on osteoporosis and previous studies.^28,29,30^

Covariates included racial and ethnic group as reported by patients (Asian, Black, White, other, and unknown), patient-level deprivation score, Charlson Comorbidity Index, smoking history, and heavy alcohol use.^31,32,33^ The deprivation score was formed by reversing CPRD quintiles to be consistent with definitions of IMD quintiles (1 = most deprived), providing an area-level measure of approximately 1500 people and a composite score across 7 domains, such as income, employment, education, etc. Any history of other eye disease, osteoporosis, fractures, and falls before the index date was also included.

We examined specific medication groups known to increase the risk of falls and fracture risk, including benzodiazepines, antidepressants, cardiovascular drugs (including antihypertensives and α-blockers), antidiabetes drugs (with a separate category for insulin), anticholinergics (only those with anticholinergic burden 2 or 3 were included, from Richardson et al^34^), and systemic steroids.^35,36^ These medication group covariates were measured before the index diagnosis date, including any history within 12 months prior, and treated as binary variables.

### Statistical Analyses

Incident rates are presented in age-standardized rates per 100 000 population using the Global Burden of Diseases population structure.^37^ Multivariable Cox proportional hazards regression was used to estimate the risk of incident falls and incident fractures (constituting events within separate models) in cases compared with comparators and to examine associations with covariates. To account for our matched cohort design, all Cox models were stratified by matched sets. The proportional hazards assumption was assessed visually via log-log plots. Missing data for IMD and race and ethnicity were coded by creating a missing category. Missing smoking status was imputed with the multivariate imputation by a chained-equations algorithm using 10 complete data sets.

Sensitivity analyses were performed using propensity scores to account for covariate imbalance between the cohorts within further Cox proportional hazard models.^38^ Variables related to each outcome at *P* < .05 were selected for inclusion in propensity weights, identified through various regression analyses with each covariate.^39^ Inverse-weighted probability models included more than 50 confounders covering demographics, long-term conditions, falls-risk–inducing medications, eye medications, and interaction terms for composite measures such as Charlson Comorbidity Index. Further sensitivity analyses were also performed for populations with a single eye disease only (ie, without concurrent history of the other eye diseases) to assess effects on outcomes. We also performed an additional analysis by separating populations for CPRD GOLD and Aurum to observe any differences between the databases (eTable 7 in Supplement 1).

Data were analyzed from May 2021 to June 2023. All analyses were performed using Stata version 16.1 (StataCorp). All *P* values were 2-sided, and there were no adjustments for multiple analyses.

## Results

### Baseline Characteristics

The delineation of each study cohort is specified in Figure 1 with baseline characteristics described in Table 1. All cohorts mainly consisted of older adults, with a mean (SD) age of 74.3 (11.5) years (mean [SD] ages of 73.8 [11.0] years, 79.4 [9.4] years, and 69.8 [13.1] years for cataract, AMD, and glaucoma, respectively) and a slightly higher proportion of females (57.2%, 62.2%, and 51.9% respectively). The cataract cohort included 410 476 cases to 2 034 194 comparators and a median (IQR) follow-up of 4.02 years (2.07-6.86 years) in cases vs 3.91 years (1.97-6.86 years) in comparators. The AMD cohort had 75 622 cases to 375 548 comparators and a median (IQR) follow-up of 3.76 years (1.91-6.49 years) in cases vs 3.61 years (1.76-6.45 years) in comparators. The glaucoma cohort consisted of 90 177 cases to 448 179 comparators and a median (IQR) follow-up of 4.46 years (2.29-7.55 years) in cases vs 4.24 (2.10-7.36 years) in controls.

**Table 1.** Baseline Characteristics for Matched Study Cohorts

Characteristic	Cataract, No. (%)		AMD, No. (%)		Glaucoma, No. (%)	
	Cases (n = 410 476)	Controls (n = 2 034 194)	Cases (n = 75 622)	Controls (n = 375 548)	Cases (n = 90 177)	Controls (n = 448 179)
Follow-up, median (IQR), y	4.0 (2.1-6.9)	4.0 (2.0-6.9)	3.8 (1.9-6.5)	3.6 (1.8-6.5)	4.5 (2.3-7.6)	4.2 (2.1-7.4)
Age, mean (SD)	73.8 (11.0)	73.8 (11.0)	79.4 (9.6)	79.3 (9.5)	69.8 (13.1)	69.8 (13.1)
Age category, y						
18-44	6703 (1.6)	33 352 (1.6)	311 (0.4)	1553 (0.4)	3615 (4.0)	17 968 (4.0)
45-64	65 949 (16.1)	328 174 (16.1)	5136 (6.8)	25 597 (6.8)	24 261 (26.9)	120 662 (26.9)
≥65	337 824 (82.3)	1 672 668 (82.2)	70 175 (92.8)	348 398 (92.8)	62 301 (69.1)	309 549 (69.1)
Deaths (all causes)	78 570 (19.1)	362 517 (17.8)	19 601 (25.9)	97 822 (26.1)	13 125 (14.6)	68 063 (15.2)
Sex						
Male	176 018 (42.9)	871 002 (42.8)	28 699 (38.0)	142 113 (37.8)	43 425 (48.2)	215 681 (48.1)
Female	234 458 (57.1)	1 163 192 (57.2)	46 923 (62.1)	233 435 (62.2)	46 752 (51.8)	232 498 (51.9)
Race and ethnicity^a^						
Asian	16 377 (4.0)	36 130 (1.8)	1103 (1.5)	4999 (1.3)	3370 (3.7)	10 799 (2.4)
Black	10 227 (2.5)	28 789 (1.4)	400 (0.5)	3569 (1.0)	4151 (4.6)	8544 (1.9)
White	362 322 (88.3)	1 631 553 (80.2)	71 009 (93.9)	325 072 (86.6)	75 233 (83.4)	346 985 (77.4)
Other	2660 (0.7)	20 887 (1.0)	290 (0.4)	2745 (0.7)	697 (0.8)	5456 (1.2)
Unknown	18 890 (4.6)	316 835 (15.6)	2820 (3.7)	39 163 (10.4)	6726 (7.5)	76 395 (17.1)
IMD						
1 (Most deprived)	16 (15.8)	305 975 (15.0)	11 061 (14.6)	54 872 (14.6)	13 723 (15.2)	67 583 (15.1)
2	74 740 (18.2)	367 573 (18.1)	13 306 (17.6)	65 705 (17.5)	16 150 (17.9)	80 710 (18.0)
3	82 422 (20.1)	413 527 (20.3)	15 276 (20.2)	75 869 (20.2)	17 774 (19.7)	90 730 (20.2)
4	88 259 (21.5)	444 586 (21.9)	16 933 (22.4)	84 500 (22.5)	19 803 (22.0)	97 602 (21.8)
5 (Least deprived)	100 036 (24.4)	500 431 (24.6)	18 999 (25.1)	94 200 (25.1)	22 679 (25.2)	111 189 (24.8)
Unknown	246 (0.1)	2102 (0.1)	47 (0.1)	402 (0.1)	48 (0.1)	365 (0.1)
CCI category						
None	177 983 (43.4)	1 827 218 (89.8)	27 541 (36.4)	304 401 (81.1)	48 675 (54.0)	396 212 (88.4)
Mild (1-2)	142 315 (34.7)	124 426 (6.1)	27 307 (36.1)	40 436 (10.8)	27 137 (30.1)	30 439 (6.8)
Moderate (3-4)	64 973 (15.8)	60 543 (3.0)	14 639 (19.4)	21 950 (5.8)	10 463 (11.6)	15 280 (3.4)
Severe (≥5)	25 205 (6.1)	22 007 (1.1)	6135 (8.1)	8761 (2.3)	3902 (4.3)	6248 (1.4)
Previous eye disease						
Cataract	NA	NA	49 405 (65.3)	148 755 (39.6)	39 446 (43.7)	109 983 (24.5)
AMD	41 039 (10.0)	91 816 (4.5)	NA	NA	6481 (7.2)	20 666 (4.6)
Glaucoma	50 191 (12.2)	112 015 (5.5)	9796 (13.0)	33 364 (8.9)	NA	NA
Medical history^b^						
Cardiovascular disease	107 768 (26.3)	332 275 (16.3)	23 450 (31.0)	85 065 (22.7)	17 176 (19.1)	68 383 (15.3)
Hypertension	171 979 (41.9)	159 275 (7.8)	35 667 (47.2)	54 921 (14.6)	32 218 (35.7)	38 607 (8.6)
Type 1 diabetes	13 149 (3.2)	7927 (0.4)	2325 (3.1)	3385 (0.9)	1954 (2.2)	2515 (0.6)
Type 2 diabetes	84 438 (20.6)	53 253 (2.6)	13 916 (18.4)	19 924 (5.3)	13 226 (14.7)	17 342 (3.9)
Asthma/COPD	19 532 (1.0)	58 932 (14.4)	8260 (2.2)	8189 (10.8)	6940 (1.6)	9259 (10.3)
Neurological condition	3132 (0.2)	9687 (2.4)	1279 (0.3)	1268 (1.7)	1052 (0.2)	1854 (2.1)
Liver disease	677 (0.0)	2783 (0.7)	218 (0.1)	322 (0.4)	266 (0.1)	377 (0.4)
Kidney disease	22 532 (1.1)	53 606 (13.1)	11 747 (3.1)	9744 (12.9)	7655 (1.7)	6977 (7.7)
Thyroid disease	11 547 (0.6)	29 534 (7.2)	4597 (1.2)	4457 (5.9)	3331 (0.7)	4499 (5.0)
Connective tissue disease	15 125 (0.7)	40 633 (9.9)	5789 (1.5)	5584 (7.4)	4710 (1.1)	6213 (6.9)
Cancer	21 718 (1.1)	51 756 (12.6)	9594 (2.6)	8543 (11.3)	6777 (1.5)	7898 (8.8)
Mental health condition	34 608 (1.7)	95 158 (23.2)	12 586 (3.4)	12 609 (16.7)	10 109 (2.3)	17 601 (19.5)
Dementia	2706 (0.1)	6342 (1.6)	2794 (0.7)	1387 (1.8)	1424 (0.3)	1024 (1.1)
Osteoporosis	41 810 (10.2)	40 356 (2.0)	10 375 (13.7)	16 829 (4.5)	6716 (7.5)	10 374 (2.3)
Current smoker	207 660 (50.6)	901 758 (44.3)	39 467 (52.2)	153 449 (40.9)	42 365 (47.0)	200 560 (44.8)
Heavy alcohol use	27 176 (6.6)	17 735 (0.9)	4107 (5.4)	4847 (1.3)	5550 (6.15)	4928 (1.1)
Fall history	88 742 (21.6)	138 892 (6.8)	20 922 (27.7)	47 497 (12.7)	15 331 (17.0)	31 667 (7.1)
Fracture history	44 827 (10.9)	110 346 (5.4)	9931 (13.1)	32 152 (8.6)	7909 (8.8)	22 277 (5.0)
Medication use						
Antidepressants	86 303 (21.0)	95 502 (4.7)	14 827 (19.6)	20 216 (5.4)	14 950 (16.6)	15 047 (3.4)
Benzodiazepines	25 906 (6.3)	20 725 (1.0)	5506 (7.3)	7121 (1.9)	4745 (5.3)	5161 (1.2)
Antihypertensives	269 556 (65.7)	235 195 (11.6)	54 024 (71.4)	81 673 (21.8)	48 741 (54.1)	59 648 (13.3)
Antidiabetes drugs	67 377 (16.4)	39 851 (2.0)	10 360 (13.7)	14 809 (3.9)	10 189 (11.3)	13 362 (3.0)
Insulin	21 003 (5.1)	8932 (0.4)	2870 (3.8)	3801 (1.0)	3326 (3.7)	3750 (0.8)
Systemic steroids	46 593 (11.4)	32 189 (1.6)	8217 (10.9)	11 721 (3.1)	7376 (8.2)	8665 (1.9)
High ACB drugs (ACB 2 or 3)	76 843 (18.7)	59 042 (2.9)	14 689 (19.4)	20 722 (5.5)	14 352 (15.9)	15 502 (3.5)

Abbreviations: ACB, anticholinergic burden; AMD, age-related macular degeneration; CCI, Charlson Comorbidity Index; COPD, chronic obstructive pulmonary disease; IMD, Index of Multiple Deprivation 2015; NA, not applicable.

^a^ The race and ethnicity category other includes patients who reported races or ethnicities that were not Asian, Black, or White.

^b^ Medical history has been grouped for ease of presentation. Cardiovascular disease includes coronary heart disease, heart failure, peripheral vascular disease, and cerebrovascular disease. Neurological condition includes epilepsy, multiple sclerosis, and Parkinson disease. Mental health condition includes anxiety, depression, eating disorder, bipolar disorder, and schizophrenia. Connective tissue disease includes rheumatoid arthritis, psoriatic arthritis, polyarthritis, and spondyloarthropathies.

At baseline, all eye disease populations had poorer health and a higher level of comorbidity, including a greater prevalence of all multiple long-term conditions compared with comparators in both physical health (eg, cardiovascular disease, respiratory conditions, osteoporosis) and mental health diagnoses (eg, depression, bipolar, dementia). This finding was also reflected in medication usage, with notably higher proportions of cases taking benzodiazepines, antidepressants, antihypertensives, antidiabetes medications, systemic steroids, and medications with a high anticholinergic burden. A higher proportion of people with eye disease had a history of both falls (approximately 3-fold for cataract and 2-fold for AMD and glaucoma) and fractures. People with eye disease were also more likely to have a history of the other 2 eye diseases compared with comparators.

### Incident Falls and Incident Fractures

During the study period, there was an increased incidence of both falls and fractures experienced by people with eye disease compared with their matched comparators. eTable 2 in Supplement 1 shows the proportions and crude rates for the primary and secondary outcomes, and eTable 3 in Supplement 1 shows the age-standardized incidence rates. Overall, a greater proportion of people with eye disease compared with comparators experienced falls (cataract, 29.7% vs 13.9%; AMD, 37.1% vs 20.7%; glaucoma, 25.0% vs 12.8%) and fractures (cataract, 14.4% vs 8.2%; AMD, 17.8% vs 11.6%; glaucoma, 12.2% vs 7.3%). The age-standardized incidence rates per 100 000 person-years for falls were 2217.5 (95% CI, 2144.5-2296.1) cases for individuals with cataract compared with 625.0 (95% CI, 611.2-639.6) in comparators, 2551.4 (95% CI, 2246.8-2956.6) in those with AMD compared with 848.1 (95% CI, 788.7-927.2) in comparators, and 1802.0 (95% CI, 1708.8-1903.8) for glaucoma compared with 621.3 (95% CI, 601.0-643.3) in comparators.

**Figure 2.**
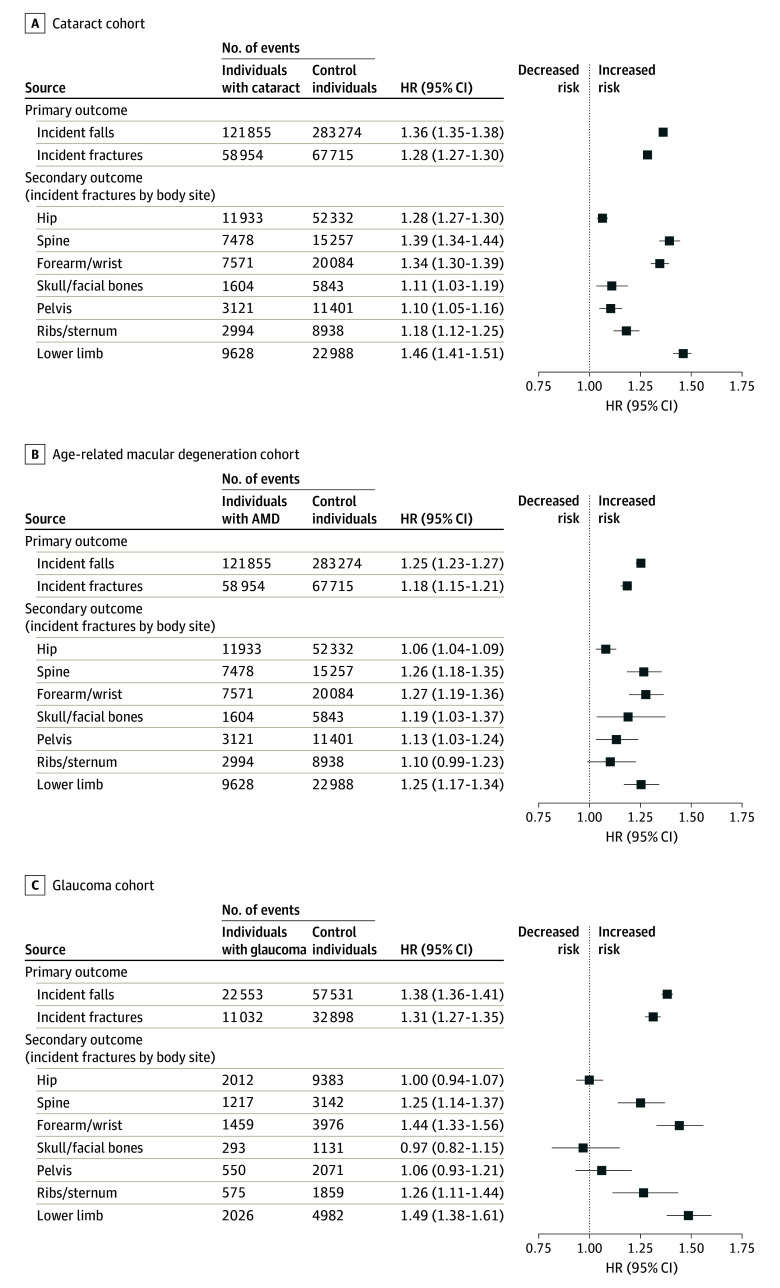
Multivariable Hazard Ratios (HRs) for Falls, Fractures, and Body Site–Specific Fractures Multivariable-adjusted HRs for primary and secondary outcomes in individuals with cataract, age-related macular degeneration (AMD), or glaucoma compared with matched comparators without eye disease.

Table 2 and Table 3 show the results of the multivariable Cox regression analysis for falls and fractures, with Figure 2 illustrating a comparison of the adjusted hazard ratios (HRs) for each eye disease cohort. Overall, there was an increased risk of falls in those with cataract (HR, 1.36; 95% CI, 1.35-1.38), AMD (HR, 1.25; 95% CI, 1.23-1.27), and glaucoma (HR, 1.38; 92% CI, 1.35-1.41) compared with matched comparators. Likewise for fractures, there also was an increased risk in all eye diseases, with an HR of 1.28 (95% CI, 1.27-1.30) in the cataract cohort, HR of 1.18 (95% CI, 1.15-1.21) for AMD, and HR of 1.31 (95% CI, 1.27-1.35) for glaucoma. As observed, HRs were slightly higher for glaucoma and cataract compared with AMD for both falls and fractures.

**Table 2.** Multivariable Hazard Ratios for Incident Falls With Adjustment for Covariates

Covariate	HR (95% CI)		
	Cataract	AMD	Glaucoma
Case	1.36 (1.35-1.38)	1.25 (1.23-1.27)	1.38 (1.36-1.41)
Age^a^	NA	NA	NA
Sex^a^	NA	NA	NA
Race^b^			
Asian	0.86 (0.83-0.88)	0.82 (0.76-0.89)	0.80 (0.75-0.86)
Black	0.59 (0.57-0.62)	0.59 (0.54-0.65)	0.60 (0.56-0.65)
Other	0.73 (0.69-0.77)	0.66 (0.59-0.74)	0.70 (0.63-0.78)
Unknown	0.23 (0.23-0.24)	0.27 (0.26-0.29)	0.25 (0.24-0.26)
IMD^b^			
2	0.94 (0.93-0.96)	0.96 (0.93-0.99)	0.94 (0.91-0.97)
3	0.90 (0.89-0.92)	0.92 (0.90-0.95)	0.88 (0.85-0.91)
4	0.88 (0.87-0.90)	0.91 (0.88-0.94)	0.85 (0.82-0.88)
5 (Least deprived)	0.86 (0.85-0.88)	0.88 (0.86-0.91)	0.84 (0.81-0.87)
Unknown	0.90 (0.78-1.05)	0.78 (0.59-1.05)	1.02 (0.72-1.45)
Previous eye disease			
Cataract	NA	1.25 (1.23-1.27)	1.31 (1.28-1.33)
AMD	1.07 (1.06-1.09)	NA	1.05 (1.02-1.08)
Glaucoma	1.16 (1.15-1.18)	1.12 (1.10-1.15)	NA
CCI score^b^			
1	1.40 (1.37-1.42)	1.30 (1.26-1.34)	1.34 (1.30-1.39)
2	1.28 (1.26-1.29)	1.18 (1.15-1.22)	1.22 (1.18-1.26)
3	1.37 (1.34-1.39)	1.30 (1.26-1.34)	1.33 (1.28-1.39)
4	1.37 (1.34-1.40)	1.30 (1.25-1.35)	1.33 (1.26-1.39)
5	1.45 (1.41-1.49)	1.34 (1.28-1.41)	1.34 (1.26-1.43)
6	1.47 (1.42-1.53)	1.43 (1.34-1.53)	1.54 (1.42-1.67)
7	1.60 (1.51-1.70)	1.66 (1.50-1.84)	1.38 (1.21-1.56)
8	1.72 (1.57-1.90)	1.65 (1.41-1.93)	2.16 (1.78-2.61)
9	1.80 (1.57-2.08)	1.68 (1.31-2.15)	2.46 (1.80-3.36)
10	1.97 (1.53-2.54)	2.02 (1.30-3.14)	2.10 (1.29-3.41)
11	2.39 (1.64-3.48)	0.92 (0.46-1.86)	1.57 (0.77-3.22)
≥12	1.84 (1.16-2.91)	1.28 (0.58-2.79)	1.84 (1.17-2.91)
Medical history			
Current smoker	1.07 (1.04-1.10)	1.04 (1.00-1.10)	1.00 (0.93-1.08)
Heavy alcohol use	1.42 (1.38-1.45)	1.24 (1.18-1.31)	1.40 (1.33-1.48)
Osteoporosis	1.50 (1.48-1.52)	1.46 (1.42-1.50)	1.48 (1.43-1.53)
Fall history	1.80 (1.78-1.82)	1.75 (1.72-1.79)	1.83 (1.79-1.88)
Medication use			
Antidepressants	1.27 (1.25-1.28)	1.32 (1.29-1.36)	1.38 (1.33-1.43)
Benzodiazepines	1.20 (1.18-1.23)	1.17 (1.13-1.22)	1.12 (1.07-1.17)
Antihypertensives	1.31 (1.30-1.32)	1.28 (1.26-1.31)	1.23 (1.20-1.26)
Antidiabetes drugs	1.18 (1.16-1.20)	1.12 (1.09-1.15)	1.17 (1.13-1.22)
Insulin	1.34 (1.30-1.38)	1.25 (1.18-1.32)	1.37 (1.29-1.46)
ACB-2 drugs	1.58 (1.51-1.66)	1.47 (1.35-1.61)	1.78 (1.61-1.98)
ACB-3 drugs	1.19 (1.17-1.21)	1.11 (1.08-1.15)	1.15 (1.11-1.19)
Systemic steroids	1.25 (1.23-1.27)	1.17 (1.13-1.21)	1.24 (1.19-1.29)

Abbreviations: ACB, anticholinergic burden; AMD, age-related macular degeneration; CCI, Charlson Comorbidity Index; HR, hazard ratio; IMD, index of multiple deprivation; NA, not applicable.

^a^ As cases and controls were matched on age and sex, there were no differences between the 2 groups in the Cox models.

^b^ Race categories were compared with White, IMD compared with 1 (most deprived), and CCI score compared with 0.

**Table 3.** Multivariable Hazard Ratios for Incident Fractures With Adjustment for Covariates

Covariate	HR (95% CI)		
	Cataract	AMD	Glaucoma
Case	1.28 (1.27-1.30)	1.18 (1.15-1.21)	1.31 (1.27-1.35)
Age^a^	NA	NA	NA
Sex^a^	NA	NA	NA
Race^b^			
Asian	0.74 (0.71-0.78)	0.87 (0.78-0.97)	0.67 (0.61-0.73)
Black	0.34 (0.32-0.36)	0.35 (0.29-0.41)	0.38 (0.34-0.43)
Other	0.71 (0.67-0.76)	0.58 (0.50-0.68)	0.63 (0.55-0.73)
Unknown	0.20 (0.20-0.21)	0.22 (0.20-0.23)	0.21 (0.20-0.23)
IMD^b^			
2	0.96 (0.94-0.98)	0.95 (0.91-0.98)	0.96 (0.92-1.00)
3	0.91 (0.90-0.93)	0.94 (0.90-0.97)	0.91 (0.87-0.95)
4	0.89 (0.87-0.91)	0.92 (0.88-0.96)	0.86 (0.82-0.90)
5 (Least deprived)	0.88 (0.86-0.90)	0.90 (0.86-0.94)	0.85 (0.81-0.89)
Unknown	0.94 (0.78-1.14)	0.81 (0.56-1.18)	0.82 (0.51-1.30)
Previous eye disease			
Cataract	NA	1.11 (1.08-1.13)	1.20 (1.17-1.23)
AMD	0.99 (0.98-1.01)	NA	1.00 (0.96-1.04)
Glaucoma	1.07 (1.05-1.08)	1.03 (1.00-1.06)	NA
CCI score^b^			
1	1.23 (1.21-1.26)	1.18 (1.13-1.22)	1.22 (1.16-1.27)
2	1.15 (1.13-1.17)	1.07 (1.03-1.11)	1.07 (1.03-1.12)
3	1.21 (1.18-1.24)	1.14 (1.09-1.20)	1.13 (1.07-1.19)
4	1.22 (1.19-1.26)	1.18 (1.12-1.24)	1.16 (1.08-1.24)
5	1.23 (1.18-1.28)	1.11 (1.03-1.19)	1.09 (1.00-1.20)
6	1.28 (1.21-1.35)	1.25 (1.13-1.37)	1.20 (1.06-1.35)
7	1.38 (1.27-1.51)	1.32 (1.14-1.52)	1.13 (0.94-1.36)
8	1.60 (1.40-1.82)	1.36 (1.08-1.71)	1.55 (1.16-2.05)
9	1.48 (1.22-1.80)	1.66 (1.15-2.37)	2.11 (1.35-3.28)
10	2.36 (1.71-3.28)	1.41 (0.74-2.70)	1.81 (0.89-3.66)
11	2.03 (1.21-3.40)	1.52 (0.64-3.61)	1.32 (0.48-3.62)
≥12	1.13 (0.56-2.26)	0.64 (0.17-2.47)	1.13 (0.56-2.26)
Medical history			
Current smoker	1.07 (1.04-1.10)	1.02 (0.96-1.10)	1.03 (0.94-1.14)
Heavy alcohol use	1.52 (1.47-1.57)	1.25 (1.17-1.34)	1.44 (1.34-1.55)
Osteoporosis	1.52 (1.49-1.55)	1.49 (1.44-1.54)	1.57 (1.50-1.65)
Fracture history	1.97 (1.93-2.00)	1.80 (1.74-1.86)	2.02 (1.94-2.11)
Medication use			
Antidepressants	1.23 (1.21-1.25)	1.25 (1.20-1.30)	1.34 (1.28-1.41)
Benzodiazepines	1.14 (1.10-1.17)	1.13 (1.07-1.18)	1.06 (1.00-1.13)
Antihypertensives	1.01 (1.00-1.03)	1.02 (1.00-1.05)	1.02 (0.98-1.05)
Antidiabetes drugs	1.06 (1.04-1.09)	1.02 (0.98-1.07)	1.06 (1.00-1.11)
Insulin	1.45 (1.39-1.51)	1.35 (1.24-1.46)	1.52 (1.39-1.66)
ACB-2 drugs	1.51 (1.42-1.61)	1.43 (1.28-1.60)	1.61 (1.40-1.84)
ACB-3 drugs	1.09 (1.07-1.11)	1.03 (0.99-1.08)	1.07 (1.02-1.12)
Systemic steroids	1.27 (1.24-1.30)	1.20 (1.15-1.25)	1.30 (1.24-1.37)

Abbreviations: ACB, anticholinergic burden; AMD, age-related macular degeneration; CCI, Charlson Comorbidity Index; HR, hazard ratio; IMD, index of multiple deprivation; NA, not applicable.

^a^ As cases and controls were matched on age and sex, there were no differences between the 2 groups in the Cox models.

^b^ Race categories were compared with White, IMD compared with 1 (most deprived), and CCI score compared with 0.

Results of our sensitivity analyses are presented in eTables 4 and 5 in Supplement 1. The inverse probability treatment weight models showed very similar HRs compared with standard Cox models. Further increased HRs were observed for both falls and fractures for all cases with single eye disease (ie, without history of concurrent eye disease) compared with comparators. For falls, the risks in the cataract-only group showed an HR of 1.44 (95% CI, 1.43-1.46); for AMD only, an HR of 1.91 (95% CI, 1.78-2.05); and for glaucoma only, an HR of 2.40 (95% CI, 2.28-2.54). For fractures, the risks for cataract only were an HR of 1.36 (95% CI, 1.33-1.38); for AMD only, an HR of 1.72 (95% CI, 1.57 - 1.88); and for glaucoma only, an HR of 2.17 (95% CI, 2.02-2.33).

### Incident Fractures by Body Site

The results of the multivariable Cox regression analysis for body site–specific fractures are shown in Figure 2 and eTable 6 in Supplement 1. Overall, all populations with eye disease had an increased risk of fractures of almost all body sites (including hip, spine, forearm, skull or facial bones, pelvis, ribs or sternum, and lower leg fractures) compared with comparators. The exceptions were for hip, pelvic, and skull and facial bone fractures in the glaucoma cohort and rib and sternal fractures for the AMD cohort, where no differences were found.

## Discussion

In this large population-based cohort study, we observed an increased risk of both falls and fractures for people with cataract, AMD, or glaucoma. This took into account a higher level of comorbidity in the population with eye disease, including multiple long-term conditions and increased medication usage. The highest effect sizes of covariates observed were higher levels of comorbidity, though not the extremes (ie, Charlson Comorbidity Index ≥12), or a history of fall or fracture. Site-specific analyses revealed an increased risk of almost all body sites, with particularly high risks for forearm and lower leg fractures. Our findings further build the evidence base demonstrating that all 3 eye diseases are important risk factors for falls and fractures. Our sensitivity analyses reported a further elevated risk within subgroup analyses of participants with single eye diseases only. Correspondingly, we found a higher risk of falls resulting in injury and have demonstrated an increased risk of both higher-impact (eg, skull and facial bones or pelvic fractures) and lower-impact fractures (eg, spinal fractures) via site-specific analyses. These findings contribute observational evidence supporting higher risks of falls or injuries for these populations and suggests a need to assess the medical and rehabilitation needs of at-risk individuals in future research.

Our findings contrast earlier cross-sectional studies reporting that only certain eye diseases are significant predictors of falls, although these all had self-reported falls outcomes that were potentially subject to recall bias.^40^ For example, a cross-sectional survey including 3280 older adults in East Asia reported a 4-fold increase in odds of falling in those with glaucoma, 1.5 times for cataract, but only 0.3 times for AMD. However, other small observation studies in France and Canada have found almost twice the risk of injurious falls in people with AMD.^41,42^ In practice, our HRs represent an increased risk over the study duration (median follow-up was approximately 4 years) but does not guarantee that the relative risk remains constant over this time. Further evidence is still needed examining the subsequent effect of increased risk of falls and fractures, potentially through examining linked outcomes such as related hospitalization and reductions in quality of life or quantifying financial effects through economic analyses. This may help further define which patients are particularly higher risk and need to access fall services and treatments more urgently.

### Limitations

Although drawing from routine electronic health data allowed a large sample size, its retrospective nature is limited by imperfect data relying on coding and irregular follow-up. There is likely a small proportion of misclassification bias, as patients who have eye disease but have yet to be assessed by an ophthalmologist (eg, early cataracts or waiting lists) may have been misclassified as not having disease. Also, we were unable to assess visual function objectively within the analysis or whether the diagnosis was monocular or binocular. Furthermore, a key limitation is that we were unable to examine treatments during follow-up because of potential inaccuracies in coding, such as cataract surgery or medication for AMD or glaucoma, which may have overestimated our HRs. This is particularly the case for the cataract cohort, where surgery can restore normal vision promptly, with cases having artificially longer follow-up. Yet this may still carry subsequent risks, including posttreatment risks such as spectacle imbalance and posterior capsule opacification. For these reasons, our analyses may be more reflective of the typical patient experience, as we theorized that a diagnosis of any eye disease may be itself a marker of increased risk.

Previous studies have reported good validity in the reporting of fractures, but there remains potential biases for our outcomes.^43,44,45,46^ As we captured all-cause traumatic fractures, a proportion may have not related to falls, but we were unable to ascertain whether this was the case. Despite this, our analysis for fractures is likely to be more accurate than for falls because of the multifactorial nature of falls. First, there may be an underestimation of falls risk as people may only present having sustained a serious fall, with injurious falls more likely to be coded. Second, other factors affecting exposure to falls risk, such as cognitive status and physical activity, were not explicitly considered, although dementia and multiple comorbidities were further adjusted for within the inverse proportional treatment weight models, which indicated very similar hazard ratios. Younger, more physically active people may have a greater risk, and though increasing comorbidity generally increases risk, very high levels of comorbidity may actually limit activity as may be observed implicitly in our reduced effect sizes of extreme Charlson Comorbidity Index scores.

## Conclusions

The results of this study indicate that people with cataract, AMD, or glaucoma have a higher risk of both falls or fractures compared with people without these eye diseases. These populations would likely benefit from improved advice, access, and referrals to falls prevention services and targeted interventions to prevent related adverse outcomes.

## Notes

Supplement 1. **eTable 1.** Codes for identifying eye diseases **eTable 2.** Proportions and crude rates of primary and secondary outcomes **eTable 3.** Incidence rates of falls and fractures (age standardized per 100,000 person years) **eTable 4.** Sensitivity analyses results tables (inverse proportional treatment weight models **eTable 5.** Sensitivity analysis 2 (single eye disease subanalysis) **eTable 6.** Multivariate-adjusted hazard ratios for incident fractures by body site with covariates **eTable 7.** Additional analysis (CPRD Aurum only analyses)

Supplement 2. Data sharing statement
