# Supplementary material for: Risk of Falls and Fractures in Individuals With Cataract, Age-Related Macular Degeneration, or Glaucoma
Source: JAMA Ophthalmol. 2023 Dec 28;142(2):96–106. doi: 10.1001/jamaophthalmol.2023.5858 (PMC10870181; doi:10.1001/jamaophthalmol.2023.5858)
Supplement: Supplement 1. — eTable 1. Codes for identifying eye diseases eTable 2. Proportions and crude rates of primary and secondary outcomes eTable 3. Incidence rates of falls and fractures (age standardized per 100,000 person years) eTable 4. Sensitivity analyses results tables (inverse proportional treatment weight models eTable 5. Sensitivity analysis 2 (single eye disease subanalysis) eTable 6. Multivariate-adjusted hazard ratios for incident fractures by body site with covariates eTable 7. Additional analysis (CPRD Aurum only analyses) [file jamaophthalmol-e235858-s001.pdf]

Supplemental Online Content

Tsang JY, Wright A, Carr MJ, et al. Risk of falls and fractures in individuals with cataract, age-related macular degeneration, or glaucoma. *JAMA Ophthalmol*. Published online December 28, 2023. doi:10.1001/jamaophthalmol.2023.5858

eTable 1. Codes for identifying eye diseases
eTable 2. Proportions and crude rates of primary and secondary outcomes
eTable 3. Incidence rates of falls and fractures (age standardized per 100,000 person years)
eTable 4. Sensitivity analyses results tables (inverse proportional treatment weight models
eTable 5. Sensitivity analysis 2 (single eye disease subanalysis)
eTable 6. Multivariate-adjusted hazard ratios for incident fractures by body site with covariates
eTable 7. Additional analysis (CPRD Aurum only analyses)

This supplemental material has been provided by the authors to give readers additional information about their work.

© 2023 Tsang JY et al. *JAMA Ophthalmology.*

eTable 1. Codes for identifying eye diseases

Read code	Aurum medcode	GOLD medcode	Read term	Eye disease
	907841000006110		[rfc] glaucoma	glaucoma
FyuG.00	388091000006115	52888	[x]glaucoma	glaucoma
FyuG100	388101000006114	98647	[x]glaucoma in endocrine,nutritional+metabolic diseases ce	glaucoma
FyuG200	299484014		[x]glaucoma in other diseases classified elsewhere	glaucoma
FyuG000	299482013	70195	[x]other glaucoma	glaucoma
F452200	459721000006110	28536	acute primary angle-closure glaucoma	glaucoma
F452300	556201000006114	35446	chronic primary angle-closure glaucoma	glaucoma
F452.11	560611000006117	6315	closed angle glaucoma	glaucoma
F45..00	40268016	2074	glaucoma	glaucoma
F404211	802901000006112	44338	glaucoma - absolute	glaucoma
F455.00	802911000006110	54262	glaucoma associated with disorders of the lens	glaucoma
F455z00	298060018	34354	glaucoma associated with disorders of the lens nos	glaucoma
F456.00	298061019	53127	glaucoma associated with other ocular disorders	glaucoma
F456z00	298071017	53521	glaucoma associated with other ocular disorders nos	glaucoma
F454000	298054018	69195	glaucoma due to chamber angle anomaly	glaucoma
F454.00	298053012	41854	glaucoma due to disease ec	glaucoma
F454z00	298059011	41804	glaucoma due to disease nos	glaucoma
F45y100	802981000006115	65193	glaucoma due to episode of increased venous pressure	glaucoma
F454100	298055017	67341	glaucoma due to iris anomaly	glaucoma
F456411	411465018	100303	glaucoma due to ocular cyst	glaucoma
F456200	803011000006118	26870	glaucoma due to ocular inflammation	glaucoma
F456500	1233026017	22528	glaucoma due to ocular trauma	glaucoma
F456400	399447011	48479	glaucoma due to ocular tumour or cyst	glaucoma
F456300	298064010	41794	glaucoma due to ocular vascular disorder	glaucoma
F454200	298056016	96707	glaucoma due to other anterior segment anomaly	glaucoma
F456100	803061000006115	37876	glaucoma due to pupillary block	glaucoma
F454300	298057013	64851	glaucoma due to systemic syndrome	glaucoma
F456000	298062014	22805	glaucoma due to unspecified ocular disorder	glaucoma
F454400	298058015	68633	glaucoma in endocrine, nutritional and metabolic diseases	glaucoma
	856601000006111		glaucoma left eye	glaucoma
F45z.00	298075014	8001	glaucoma nos	glaucoma
	856591000006115		glaucoma right eye	glaucoma
1482.00	251653013	8955	h/o: glaucoma	glaucoma
F45y000	49139016	63660	hypersecretion glaucoma	glaucoma
7259100	383381000000118	89934	injection of bleb following glaucoma surgery	glaucoma
F452100	770481000006118	44817	intermittent primary angle-closure glaucoma	glaucoma
7259400	378701000000110	93967	laser suture lysis following glaucoma surgery	glaucoma
F45y200	84105018	8132	low tension glaucoma	glaucoma
F451200	734071000006115	9469	low tension glaucoma	glaucoma
7259000	383421000000110	65079	needling of bleb following glaucoma surgery	glaucoma

© 2023 Tsang JY et al. *JAMA Ophthalmology.*

F456600	347755010	11058	neovascular glaucoma	glaucoma
F451211	495680018	53879	normal pressure glaucoma	glaucoma
F450100	264071000006115	10070	open angle glaucoma with borderline intraocular pressure	glaucoma
F451.00	140102012	1798	open-angle glaucoma	glaucoma
F451z00	298042018	28189	open-angle glaucoma nos	glaucoma
F451500	47081000006113	72394	open-angle glaucoma residual stage	glaucoma
7259.00	361511000000118	46069	operations following glaucoma surgery	glaucoma
7259z00	383401000000118	95852	operations following glaucoma surgery nos	glaucoma
F4H1400	1220514012	20230	optic disc glaucomatous atrophy	glaucoma
F45y.00	298072012	28505	other specified forms of glaucoma	glaucoma
F45yz00	298074013	44295	other specified glaucoma nos	glaucoma
7259y00	378721000000118	88142	other specified operations following glaucoma surgery	glaucoma
7275.00	249331000006112	11059	pan retinal photocoagulation for glaucoma	glaucoma
F455000	54899010	44798	phacolytic glaucoma	glaucoma
F451300	76978010	12251	pigmentary glaucoma	glaucoma
F452.00	1486268015	2823	primary angle-closure glaucoma	glaucoma
F452z00	298049010	39120	primary angle-closure glaucoma nos	glaucoma
F452400	298048019	67413	primary angle-closure glaucoma residual stage	glaucoma
F452.99	883291000006119		primary closed-angle glaucoma	glaucoma
F451100	503452018	4581	primary open-angle glaucoma	glaucoma
F455100	178757012	18743	pseudoexfoliation glaucoma	glaucoma
7259300	2676361011	91442	removal of releasable suture following glaucoma surgery	glaucoma
7259200	378061000000112	88595	revision of bleb nec following glaucoma surgery	glaucoma
F456611	347754014	9213	rubeotic glaucoma	glaucoma
F45y.99	883301000006118		secondary/other glaucoma	glaucoma
F451111	141721000006112	30649	simple chronic glaucoma	glaucoma
F453.00	1221368012	35528	steroid-induced glaucoma	glaucoma
F453000	298050010		steroid-induced glaucoma glaucomatous stage	glaucoma
F453z00	298052019	48132	steroid-induced glaucoma nos	glaucoma
F453100	298051014	68094	steroid-induced glaucoma residual stage	glaucoma
F451000	298034019	42447	unspecified open-angle glaucoma	glaucoma
F452000	298047012	20520	unspecified primary angle-closure glaucoma	glaucoma
	907861000006114		[rfc] cataract	cataract
ZV45611	1227680019	4459	[v]state following cataract extraction	cataract
FyuE400	299468013	49297	[x]cataract in other diseases classified elsewhere	cataract
FyuE300	370441000006113		[x]cataract/oth endocrine,nutritional+metabolic diseases ce	cataract
FyuE000	299464010	101939	[x]other senile cataract	cataract
FyuE100	299465011	70201	[x]other specified cataract	cataract
F465.00	473371000006112	33482	after cataract	cataract
F465z00	298137012	44487	after cataract nos	cataract
F465300	298135016	26850	after-cataract with vision obscured	cataract
F460300	3463017	59125	anterior subcapsular polar cataract	cataract
F461600	178759010	92358	anterior subcapsular polar senile cataract	cataract
F466.00	158572010	703	bilateral cataracts	cataract
P33y000	518941000006114	27898	blue dot cataract	cataract

© 2023 Tsang JY et al. *JAMA Ophthalmology.*

F4B4C00	2124371000000115	105971	bullous aphakic keratopathy following cataract surgery	cataract
F46y000	19035016	24481	calcification of lens	cataract
P331.00	313009012	59914	capsular and subcapsular cataract	cataract
P331000	313010019	18089	capsular cataract	cataract
P331z00	313011015	88738	capsular or subcapsular cataract nos	cataract
F46..00	298076010	296	cataract	cataract
F464300	298128012	58625	cataract associated with other syndromes	cataract
F464.00	298126011	71291	cataract due to other disorder	cataract
F464z00	298132018	94430	cataract due to other disorder nos	cataract
F464700	298131013		cataract due to other physical inflammation	cataract
7267399	928811000006119		cataract extract + lens implant	cataract
7267299	859791000006115		cataract extract +lens implant	cataract
	853291000006111		cataract extraction & lens repl	cataract
7267600	1479675015	103508	cataract extraction and insertion of intraocular lens	cataract
	1771661000006117		cataract extraction and insertion of intraocular lens	cataract
7266z99	859771000006116		cataract extraction unspec.	cataract
F463400	36608018	19371	cataract in degenerative disorder	cataract
F463200	298117013	54130	cataract in eye inflammatory disorder	cataract
F46z.00	298145019	6317	cataract nos	cataract
2BT..00	539421000006110	64196	cataract observation	cataract
8LC0.00	567741000000116	94348	cataract operation planned	cataract
F463.00	298113012	48228	cataract secondary to ocular disease	cataract
F463z00	298125010	46169	cataract secondary to ocular disorder nos	cataract
F463300	126733015	61325	cataract with neovascularization	cataract
F461x00	574651000006115	70257	combined senile cataract	cataract
F461200	21000016	50932	coronary cataract	cataract
P332.00	313012010	96385	cortical and zonular cataract	cataract
F460500	298083015	7257	cortical cataract	cataract
P332z00	313017016	98962	cortical or zonular cataract nos	cataract
F461800	130863017	22022	cortical senile cataract	cataract
7264200	267090015	51648	cryoextraction of lens	cataract
7266000	504155019	54975	curettage of lens	cataract
F464000	73294018	10659	diabetic cataract	cataract
F464400	630441000006118	58120	drug induced cataract	cataract
7263.12	659551000006113	6330	extracapsular extraction of cataract	cataract
7263.00	23515018	10706	extracapsular extraction of lens	cataract
7263z00	267085018	38487	extracapsular extraction of lens nos	cataract
7263.99	859701000006110		extracapsular lens extraction	cataract
7264000	267089012	24108	forceps extraction of lens	cataract
14NC.00	1489323012	11970	h/o: bilateral cataract extraction	cataract
1483.00	251654019	5518	h/o: cataract	cataract
14NA.00	451375018	28515	h/o: l cataract extraction	cataract
14N9.00	451374019	28513	h/o: r cataract extraction	cataract
F461B00	399449014	51162	hypermature cataract	cataract
F461500	298092017	23631	immature cataract nos	cataract
F46z000	455415018	44294	immature cortical cataract	cataract

© 2023 Tsang JY et al. *JAMA Ophthalmology.*

F461400	298091012	48148	incipient cataract nos	cataract
C108F00	771341000006114	44260	insulin dependent diabetes mellitus with diabetic cataract	cataract
C10EF12	913891000006113	100770	insulin dependent diabetes mellitus with diabetic cataract	cataract
7264.11	267088016	4260	intracapsular extraction of cataract	cataract
7264.00	267086017	24113	intracapsular extraction of lens	cataract
7264z00	267092011	5511	intracapsular extraction of lens nos	cataract
F4B4B00	2124391000000116	104553	keratopathy following cataract surgery	cataract
F460600	298084014	45190	lamellar zonular cataract	cataract
	853641000006116		left cataract extraction	cataract
	853711000006111		left cataract extraction and implant	cataract
7264299	859741000006112		lens cryoextraction	cataract
7266099	504156018		lens curette extraction	cataract
7264099	859721000006117		lens forceps extraction	cataract
7264199	859731000006119		lens suction extraction	cataract
7266200	267108015	31087	mechanical lensectomy	cataract
F461B11	699721000006118	63640	morgagni cataract	cataract
F464200	107602013	60883	myotonic cataract	cataract
C109E00	281161000006114	69278	non-insulin depend diabetes mellitus with diabetic cataract	cataract
F460700	89579016	7793	nuclear cataract	cataract
F461900	298096019	6876	nuclear senile cataract	cataract
22E5.00	253774010	1622	o/e - cataract present	cataract
2BT3.00	451396013	19729	o/e - left cataract absent	cataract
2BT1.00	451394011	9931	o/e - left cataract present	cataract
2BT2.00	451395012	21108	o/e - right cataract absent	cataract
2BT0.00	451393017	6547	o/e - right cataract present	cataract
F465200	298134017	89585	other after cataract with vision normal	cataract
F46y.00	298142016	4358	other cataract	cataract
F46yz00	298144015	15589	other cataract nos	cataract
7266.11	410918012	5361	other extraction of cataract	cataract
7266.00	31741000006111	1445	other extraction of lens	cataract
7266z00	267110018	14751	other extraction of lens nos	cataract
F461y00	298102014	49085	other senile cataract	cataract
7263y00	267084019	49241	other specified extracapsular extraction of lens	cataract
7264y00	267091016	45316	other specified intracapsular extraction of lens	cataract
7266y00	267109011	42702	other specified other extraction of lens	cataract
7263111	505939016	1974	phacoemulsification of lens	cataract
7263100	505940019	2301	phakoemulsification of lens	cataract
F465500	217851000006114	8130	posterior capsule opacification	cataract
F460400	57632013	4242	posterior subcapsular polar cataract	cataract
F461700	9918017	5325	posterior subcapsular polar senile cataract	cataract
F461300	67912012	26097	punctate cataract	cataract
F464600	189671000006110	66093	radiation induced cataract	cataract
8H5H.00	2159947017	11767	referral for cataract extraction	cataract
8HTV.00	2533421012	11941	referral to cataract clinic	cataract
	853651000006119		right cataract extraction	cataract
	853701000006113		right cataract extraction and implant	cataract

© 2023 Tsang JY et al. *JAMA Ophthalmology.*

F464.99	991741000006111		secondary cataract	cataract
F446499	989061000006112		secondary cataract	cataract
F463.99	883311000006115		secondary cataract	cataract
F461.00	66160017	10010	senile cataract	cataract
F461z00	298103016	29770	senile cataract nos	cataract
P331100	158573017	11255	subcapsular cataract	cataract
7264100	486604016	41493	suction extraction of lens	cataract
F464100	113312017	45952	tetanic cataract	cataract
F462200	15277015	73419	total traumatic cataract	cataract
F461A00	298097011	33793	total, mature senile cataract	cataract
F464500	298130014		toxic cataract not due to drugs	cataract
F462.00	57359011	3897	traumatic cataract	cataract
F462z00	298112019	50638	traumatic cataract nos	cataract
C10EF00	913901000006112	49554	type 1 diabetes mellitus with diabetic cataract	cataract
C108F12	84321000006114	110400	type 1 diabetes mellitus with diabetic cataract	cataract
C10FE00	914301000006111	44982	type 2 diabetes mellitus with diabetic cataract	cataract
C109E12	84501000006114	44779	type 2 diabetes mellitus with diabetic cataract	cataract
C108F11	84691000006115	17545	type i diabetes mellitus with diabetic cataract	cataract
C10EF11	913911000006110		type i diabetes mellitus with diabetic cataract	cataract
C109E11	84871000006117	48192	type ii diabetes mellitus with diabetic cataract	cataract
C10FE11	914311000006114	93727	type ii diabetes mellitus with diabetic cataract	cataract
F463000	298116016	62188	unspecified cataracta complicata	cataract
F465000	298133011	24467	unspecified secondary cataract	cataract
F461000	298090013	47566	unspecified senile cataract	cataract
F462000	298104010	62605	unspecified traumatic cataract	cataract
F4K2D00	298880018	42452	vitreous syndrome following cataract surgery	cataract
P332100	313014011	63429	zonular cataract	cataract
F425.00	399433012	118	degeneration of macula and posterior pole	age-related macular degeneration
F425z00	297816017	1753	degeneration of macula or posterior pole nos	age-related macular degeneration
F425100	2537365016	10343	dry senile macular degeneration	age-related macular degeneration
F425.11	399676018	6350	senile macular degeneration	age-related macular degeneration
F425000	78571000006118	2762	unspecified senile macular degeneration	age-related macular degeneration
F425200	2538088014	26205	wet senile macular degeneration	age-related macular degeneration

© 2023 Tsang JY et al. *JAMA Ophthalmology.*

eTable 2. Proportions and crude rates of primary and secondary outcomes

	Cataract		Age-related Macular Degeneration		Glaucoma	
	**Cases (n = 410,476)**	**Controls (n = 2,034,194)**	**Cases (n = 75,622)**	**Controls (n = 375,548)**	**Cases (n = 90,177)**	**Controls (n = 448,179)**
**Primary outcomes**						
Incident falls	29.7% (n=121,855)	13.9% (n=283,274)	37.1% (n=28,059)	20.6% (n=77,395)	25.0% (n=22,553)	12.8% (n=57,531)
Incident fractures	14.4% (n=58,954)	8.2% (n=167,715)	17.8% (n=13,454)	11.6% (n=43,623)	12.2% (n=11,032)	7.3% (n=32,898)
**Secondary outcomes (Incident fractures by body site)**						
Hip	2.9% (n=11933)	2.6% (n=52332)	4.4% (n=3288)	3.8% (n=14229)	2.2% (n=2012)	2.1% (n=9383)
Spine	1.8% (n=7478)	0.8% (n=15257)	2.4% (n=1799)	1.1% (n=4254)	1.4% (n=1217)	0.7% (n=3142)
Wrist/forearm	1.8% (n=7571)	1.0% (n=20084)	2.2% (n=1687)	1.3% (n=4950)	1.6% (n=1459)	0.9% (n=3976)
Skull/facial bones	0.4% (n=1604)	0.3% (n=5843)	0.5% (n=370)	0.3% (n=1270)	0.3% (n=293)	0.3% (n=1131)
Pelvis	0.8% (n=3121)	0.6% (n=11401)	1.2% (n=905)	0.9% (n=3360)	0.6% (n=550)	0.5% (n=2071)
Ribs/Sternum	0.7% (n=2994)	0.4% (n=8938)	0.9% (n=653)	0.6% (n=2203)	0.6% (n=575)	0.4% (n=1859)
Lower leg	2.4% (n=9628)	1.1% (n=22988)	2.4% (n=1781)	1.4% (n=5399)	2.3% (n=2026)	1.1% (n=4982)

© 2023 Tsang JY et al. *JAMA Ophthalmology.*

eTable 3. Incidence rates of falls and fractures (age standardized per 100,000 person years)

	Cataract		Age-related Macular Degeneration		Glaucoma	
	Cases (95% CI)	Controls (95% CI)	Cases (95% CI)	Controls (95% CI)	Cases (95% CI)	Controls (95% CI)
**Primary outcomes**						
Incident falls	2,217.51 (2,143.50-2,296.12)	624.97 (611.18-639.65)	2,551.39 (2,246.83-2,956.60)	848.11 (788.72-927.16)	1,802.01 (1,708.79-1,903.79)	621.27 (600.99-643.27)
Incident fractures	1,212.73 (1,155.41-1,274.53)	388.34 (376.80-400.77)	1,292.83 (1,078.77-1,604.44)	505.87 (455.66-575.73)	973.54 (902.62-1,052.83)	380.78 (363.77-399.50)
**Secondary outcomes (Incident fractures by body site)**						
Hip	102.32 (414.80-115.94)	75.27 (72.83-78.60)	139.27 (110.42-292.59)	108.35 (97.50-141.52)	73.55 (65.89-90.50)	67.20 (63.97-72.21)
Spine	91.45 (80.06-107.15)	27.63 (25.39-30.76)	74.49 (65.92-85.57)	32.27 (29.93-35.12)	63.70 (51.12-84.59)	30.43 (26.47-36.10)
Forearm	125.92 (110.54-145.58)	54.28 (49.99-59.45)	132.76 (99.39-290.80)	59.7 (48.68-93.04)	117.49 (96.41-146.74)	51.29 (115.06-136.60)
Skull/facial bones	50.62 (39.15-66.38)	26.59 (22.85-31.21)	41.77 (17.33-191.14)	26.78 (15.22-59.54)	30.93 (20.16-50.00)	24.58 (19.60-31.25)
Pelvis	26.07 (22.39-34.43)	16.54 (15.39-18.61)	32.47 (27.96-39.52)	27.76 (21.99-59.44)	18.67 (16.37-21.61)	15.86 (13.92-19.62)
Ribs/Sternum	56.50 (46.25-71.06)	20.75 (18.41-23.98)	35.60 (28.88-44.84)	32.45 (20.74-65.39)	48.90 (35.80-70.25)	22.19 (18.62-27.49)
Lower leg	330.38 (299.31-365.71)	73.52 (68.01-79.90)	259.46 (175.38-441.30)	84.97 (64.93-125.13)	285.32 (245.51-333.18)	78.59 (70.13-88.73)

© 2023 Tsang JY et al. *JAMA Ophthalmology.*

eTable 4. Sensitivity analyses results tables (inverse proportional treatment weight models

(Abbreviations: ACB = anticholinergic burden, AMD = age-related macular degeneration, CCI = Charlson Comorbidity Index, IMD = index of multiple deprivation, SD = standard deviation)

eTable 4.1 Sensitivity analysis 1 (Inverse proportional treatment weight analyses – all propensity adjusted confounders included in the analysis)

	Cataract			AMD			Glaucoma		
	Mean in 'treated'	Mean in 'un-treated'	Standardised diff/ effect size	Mean in 'treated'	Mean in 'un-treated'	Standardised diff/ effect size	Mean in 'treated'	Mean in 'untreated'	Standardised diff/ effect size
**Gender**	1.57	1.57	-0.001	1.62	1.62	-0.002	1.52	1.52	-0.001
**Age**	73.85	73.76	0.008	79.42	79.32	0.01	69.82	69.78	0.003
**Race**									
**White**	0.88	0.80	0.084	0.94	0.87	0.061	0.83	0.77	0.052
**Other**	0.01	0.01	0.019	0.00	0.01	0.085	0.01	0.01	0.003
**Asian**	0.04	0.02	0.030	0.01	0.01	0.008	0.04	0.02	0.013
**Black**	0.03	0.01	0.194	0.01	0.01	0.006	0.05	0.02	0.032
**Unknown**	0.05	0.16	0.572	0.04	0.10	0.323	0.07	0.17	0.165
**IMD**									
**1**	0.16	0.15	0.003	0.15	0.15	0.003	0.15	0.15	0.085
**2**	0.18	0.18	0.001	0.18	0.18	0.001	0.18	0.18	0.003
**3**	0.20	0.20	0.002	0.20	0.20	0.002	0.20	0.20	0.007
**4**	0.22	0.22	0.003	0.22	0.23	0.003	0.22	0.22	0.006
**5**	0.24	0.25	0.006	0.25	0.25	0.074	0.25	0.25	0.018
**Unknown**	0.01	0.01	-0.296	0.01	0.01	-0.579	0.01	0.01	-0.216
**CCI score**									
**0**	0.43	0.90	-1.149	0.36	0.81	-1.502	0.54	0.88	-0.407
**1**	0.17	0.03	0.553	0.17	0.05	0.334	0.15	0.03	0.478
**2**	0.18	0.03	0.563	0.19	0.06	0.355	0.15	0.04	0.451
**3**	0.10	0.02	0.516	0.12	0.04	0.329	0.07	0.02	0.340
**4**	0.06	0.01	0.569	0.07	0.02	0.361	0.04	0.01	0.374
**5**	0.03	0	0.060	0.05	0.01	0.436	0.02	0.01	0.163
**6**	0.02	0	0.068	0.02	0.01	0.053	0.01	0	0.018
**7**	0.01	0	0.071	0.01	0.00	0.049	0	0	0.013
**8**	0	0	0.081	0	0	0.057	0	0	0.097
**9**	0	0	0.073	0	0	0.055	0	0	0.016
**10**	0	0	0.061	0	0	0.031	0	0	0.020
**11**	0	0	0.008	0	0	0.014	0	0	
**12+**	0	0	0.007	0	0	0.147	0	0	0.094
**Previous eye disease**									
**Cataract**	N/A			0.65	0.4	0.533	0.44	0.25	0.414
**Glaucoma**	0.12	0.06	0.238	0.13	0.09	0.131	N/A		
**AMD**	0.1	0.05	0.213	N/A			0.07	0.05	0.109
**Past Medical History**									
**Cardiovascular disease**	0.26	0.16	0.244	0.31	0.23	0.189	0.19	0.15	0.101
**Hypertension**	0.42	0.08	0.858	0.47	0.15	0.752	0.36	0.09	0.691
**Diabetes**	0.44	0.06	0.627	0.40	0.12	0.444	0.32	0.08	0.403
**Asthma**	0.06	0	0.327	0.05	0.01	0.241	0.03	0.01	0.182
**COPD**	0.1	0.01	0.422	0.07	0.01	0.275	0.08	0.01	0.338
**Other respiratory**	0.14	0.01	0.521	0.11	0.02	0.355	0.1	0.02	0.376

© 2023 Tsang JY et al. *JAMA Ophthalmology.*

Cancer	0.13	0.01	0.47	0.11	0.03	0.35	0.09	0.02	0.333
Dementia	0.02	0	0.155	0.02	0.01	0.097	Omitted		
Liver disease	0.01	0	0.108	0	0	0.075	0	0	0.074
Renal disease	0.13	0.01	0.479	0.13	0.03	0.365	0.08	0.02	0.287
Thyroid disease	0.07	0.01	0.348	0.06	0.01	0.254	0.05	0.01	0.257
Neurological condition	0.02	0	0.199	0.02	0	0.134	0.02	0	0.172
Learning disability	0	0	0.04	0	0	0.017	0	0	0.036
Mental health condition	0.23	0.02	0.688	0.17	0.03	0.455	0.2	0.02	0.577
Peptic ulcer disease	0.03	0	0.224	0.03	0	0.171	0.02	0	0.163
Connective tissue disease	0.1	0.01	0.417	0.07	0.02	0.286	0.07	0.01	0.302
Osteoporosis	0.1	0.02	0.348	0.14	0.04	0.325	0.07	0.02	0.24
Prior fracture history	0.11	0.05	0.202	0.13	0.09	0.147	0.09	0.05	0.151
Prior fracture history in last year	0.03	0.01	0.097	0.03	0.02	0.067	0.02	0.01	0.07
Prior fall history	0.22	0.07	0.433	0.28	0.13	0.381	0.17	0.07	0.309
Prior fracture history in last year	0.05	0.01	0.2	0.07	0.03	0.181	0.04	0.01	0.133
Medication use									
Antidepressant	0.21	0.05	0.503	0.2	0.05	0.44	0.17	0.03	0.452
Benzodiazepine	0.06	0.01	0.284	0.07	0.02	0.26	0.05	0.01	0.235
Cardiovascular	0.66	0.12	1.337	0.71	0.22	1.149	0.54	0.13	0.955
Antidiabetic	0.16	0.02	0.517	Omitted			Omitted		
Insulin	0.05	0	0.288	Omitted			Omitted		
Systemic steroids	0.11	0.02	0.405	0.11	0.03	0.307	0.08	0.02	0.288
Oestrogen	0.01	0	0.135	0.01	0	0.088	0.02	0	0.162
Current smoker	0.09	0.02	0.095	0.10	0.03	0.085	0.07	0.02	0.068
Heavy alcohol use	0.07	0.01	0.306	0.05	0.01	0.231	0.06	0.01	0.273
Eye medication use/treatment									
Miotics	0	0	0.02	Omitted			0	0	0.046
Betablockers	0.01	0	0.092	0.01	0	0.06	0.03	0	0.233
Carbonic Anhydrase Inhibitors	0.01	0	0.101	0.01	0.01	0.058	0.03	0	0.237
Prostaglandins	0.05	0.01	0.204	0.05	0.03	0.125	0.27	0	0.85
Sympathomimetics	0.01	0	0.077	0.01	0	0.036	0.01	0	0.159
Antimuscarinic eye treatments	0	0	0.07	0	0	0.029	0.01	0	0.102
Anti-infective eye treatments	0.07	0.01	0.328	0.07	0.02	0.244	0.06	0.01	0.269
Eye anti-inflammatories	0.03	0	0.208	0.02	0.01	0.126	0.03	0	0.199
Eye lubricants	0.08	0.01	0.313	0.1	0.03	0.283	0.07	0.02	0.274
Other eye medications	0.01	0	0.083	0.01	0	0.04	0.02	0	0.178
Interaction terms									
CCI x CCI	4.94	0.89	0.541	6.11	1.79	0.492	3.66	1.08	0.376

© 2023 Tsang JY et al. *JAMA Ophthalmology.*

**CCI x cardiovascular disease**	0.67	0.11	0.485	0.85	0.25	0.438	0.46	0.14	0.305
**CCI x Diabetes**	1.17	0.17	0.456	1.24	0.35	0.356	0.81	0.24	0.283
**CCI x cancer**	0.23	0.04	0.593	0.28	0.08	0.515	0.16	0.05	0.376
**CCI x dementia**	0.33	0.04	0.517	Omitted			Omitted		
**CCI x liver disease**	Omitted			0.02	0	0.069	0.02	0	0.062
**CCI x renal disease**	0.43	0.04	0.419	0.42	0.09	0.332	0.25	0.05	0.254
**CCI x respiratory disorder**	0.27	0.02	0.358	0.24	0.05	0.26	0.15	0.03	0.208
**CCI x peptic ulcer disease**	0.09	0.01	0.185	0.08	0.01	0.144	0.05	0.01	0.125
**CCI x connective disorder**	0.2	0.02	0.296	0.17	0.04	0.215	0.11	0.02	0.181
**Cardiovascular disease x antihypertensives**	0.05	0.01	0.192	0.05	0.01	0.142	Omitted		
**Diabetes x anti-diabetic drugs**	0.04	0.01	0.202	Omitted			Omitted		
**Diabetes x oral steroids**	0.31	0.03	0.357	0.3	0.06	0.276	0.2	0.04	0.24

eTable 4.2 Sensitivity analysis 1 (Inverse proportional treatment weight models – falls)

	**Cataract**		**AMD**		**Glaucoma**	
	**HR**	**95% CI**	**HR**	**95% CI**	**HR**	**95% CI**
**Case**	1.36	(1.35 - 1.38)	1.24	(1.22 - 1.27)	1.41	(1.37 - 1.44)
**Age**	1 (Omitted)		1 (Omitted)		1 (Omitted)	
**Gender**	1 (Omitted)		1 (Omitted)		1 (Omitted)	
**Race***						
**Other**	0.72	(0.68 - 0.76)	0.67	(0.59 - 0.76)	0.70	(0.62 - 0.80)
**Asian**	0.87	(0.84 - 0.91)	0.84	(0.77 - 0.92)	0.84	(0.78 - 0.91)
**Black**	0.61	(0.59 - 0.64)	0.58	(0.52 - 0.65)	0.62	(0.57 - 0.68)
**Unknown**	0.29	(0.29 - 0.30)	0.32	(0.30 - 0.34)	0.23	(0.21 - 0.24)
**IMD***						
**2**	0.94	(0.92 - 0.95)	0.93	(0.90 - 0.96)	0.92	(0.88 - 0.95)
**3**	0.90	(0.88 - 0.91)	0.90	(0.88 - 0.93)	0.86	(0.83 - 0.90)
**4**	0.88	(0.87 - 0.90)	0.90	(0.87 - 0.93)	0.83	(0.80 - 0.87)
**5 (least deprived)**	0.86	(0.85 - 0.88)	0.86	(0.83 - 0.89)	0.81	(0.77 - 0.84)
**Unknown**	0.95	(0.80 - 1.12)	0.79	(0.57 - 1.08)	0.89	(0.59 - 1.36)
**Previous eye disease**						
**Cataract**	1 (Omitted)		1.26	(1.24 - 1.29)	1.39	(1.36 - 1.43)
**Glaucoma**	1.18	(1.16 - 1.20)	1.12	(1.09 - 1.15)	1 (Omitted)	
**AMD**	1.08	(1.06 - 1.09)	1 (Omitted)		1.05	(1.01 - 1.09)
**CCI Score***						
**1**	1.41	(1.39 - 1.43)	1.29	(1.25 - 1.33)	1.31	(1.26 - 1.36)
**2**	1.28	(1.26 - 1.30)	1.18	(1.15 - 1.21)	1.20	(1.16 - 1.25)
**3**	1.37	(1.34 - 1.40)	1.30	(1.25 - 1.34)	1.32	(1.26 - 1.39)

© 2023 Tsang JY et al. *JAMA Ophthalmology.*

4	1.35	(1.32 - 1.39)	1.29	(1.23 - 1.34)	1.32	(1.25 - 1.40)
5	1.46	(1.41 - 1.51)	1.37	(1.30 - 1.45)	1.36	(1.27 - 1.47)
6	1.54	(1.47 - 1.61)	1.43	(1.34 - 1.54)	1.55	(1.41 - 1.70)
7	1.61	(1.51 - 1.73)	1.67	(1.50 - 1.85)	1.46	(1.26 - 1.69)
8	1.71	(1.54 - 1.91)	1.64	(1.39 - 1.93)	2.31	(1.83 - 2.91)
9	1.95	(1.64 - 2.33)	1.81	(1.39 - 2.35)	2.28	(1.48 - 3.53)
10	2.29	(1.60 - 3.27)	2.08	(1.30 - 3.32)	1.97	(0.95 - 4.09)
11	2.37	(1.26 - 4.45)	0.71	(0.29 - 1.75)	1.20	(0.36 - 3.96)
12+	2.57	(0.86 - 7.72)	1.90	(0.25 - 9.41)	1.48	(0.56 - 8.78)
Current smoker	1.09	(1.05 - 1.12)	1.04	(0.98 - 1.11)	0.98	(0.89 - 1.08)
Heavy alcohol use	1.41	(1.37 - 1.45)	1.24	(1.17 - 1.31)	1.38	(1.30 - 1.48)
Medication use<br>Antidepressants	1.25	(1.23 - 1.27)	1.34	(1.29 - 1.38)	1.39	(1.33 - 1.45)
Benzodiazepines	1.21	(1.18 - 1.24)	1.16	(1.11 - 1.21)	1.14	(1.08 - 1.21)
Anti-hypertensives	1.31	(1.29 - 1.32)	1.28	(1.25 - 1.30)	1.23	(1.19 - 1.27)
Anti-diabetic drugs	1.19	(1.16 - 1.21)	1.12	(1.08 - 1.16)	1.15	(1.10 - 1.20)
Insulin	1.30	(1.25 - 1.35)	1.24	(1.17 - 1.32)	1.34	(1.24 - 1.44)
ACB-2 drugs	1.60	(1.52 - 1.70)	1.44	(1.32 - 1.58)	1.67	(1.47 - 1.89)
ACB-3 drugs	1.22	(1.20 - 1.24)	1.11	(1.08 - 1.15)	1.14	(1.10 - 1.19)
Systemic steroids	1.26	(1.24 - 1.29)	1.18	(1.14 - 1.22)	1.24	(1.18 - 1.30)
Osteoporosis	1.53	(1.50 - 1.56)	1.48	(1.44 - 1.52)	1.46	(1.40 - 1.52)
Prior fracture history	1.83	(1.80 - 1.85)	1.77	(1.73 - 1.80)	1.86	(1.80 - 1.91)

*Race compared to White, IMD compared to 1 (most deprived), CCI compared to CCI=0

eTable 4.3 Sensitivity analysis 1 (Inverse proportional treatment weight models – fractures)

	Cataract		AMD		Glaucoma	
	HR	95% CI	HR	95% CI	HR	95% CI
Case	1.28	(1.27 - 1.30)	1.17	(1.14 - 1.20)	1.31	(1.26 - 1.35)
Age	1 (Omitted)		1 (Omitted)		1 (Omitted)	
Gender	1 (Omitted)		1 (Omitted)		1 (Omitted)	
Race*						
Other	0.70	(0.65 - 0.75)	0.57	(0.47 - 0.68)	0.68	(0.58 - 0.80)
Asian	0.73	(0.69 - 0.77)	0.88	(0.78 - 0.99)	0.66	(0.59 - 0.74)
Black	0.34	(0.32 - 0.37)	0.32	(0.26 - 0.39)	0.40	(0.34 - 0.45)
Unknown	0.24	(0.23 - 0.25)	0.23	(0.21 - 0.25)	0.19	(0.18 - 0.21)
IMD*						
2	0.95	(0.93 - 0.98)	0.93	(0.89 - 0.97)	0.93	(0.88 - 0.98)
3	0.91	(0.89 - 0.93)	0.92	(0.88 - 0.96)	0.90	(0.85 - 0.94)
4	0.89	(0.87 - 0.92)	0.92	(0.88 - 0.96)	0.84	(0.80 - 0.89)
5 (least deprived)	0.87	(0.85 - 0.90)	0.90	(0.86 - 0.94)	0.82	(0.77 - 0.86)
Unknown	1.00	(0.80 - 1.24)	0.81	(0.54 - 1.22)	0.96	(0.56 - 1.65)
Previous eye disease<br>Cataract	1 (Omitted)		1.10	(1.07 - 1.13)	1.23	(1.19 - 1.27)
Glaucoma	1.08	(1.06 - 1.10)	1.02	(0.99 - 1.06)	1 (Omitted)	
AMD	1.00	(0.98 - 1.02)	1 (Omitted)		1.00	(0.96 - 1.05)

© 2023 Tsang JY et al. *JAMA Ophthalmology.*

**CCI Score***						
**1**	1.25	(1.22 - 1.27)	1.20	(1.15 - 1.25)	1.20	(1.14 - 1.27)
**2**	1.16	(1.14 - 1.19)	1.08	(1.04 - 1.12)	1.08	(1.02 - 1.13)
**3**	1.22	(1.18 - 1.25)	1.15	(1.10 - 1.21)	1.14	(1.07 - 1.22)
**4**	1.20	(1.16 - 1.24)	1.18	(1.11 - 1.25)	1.21	(1.12 - 1.30)
**5**	1.22	(1.17 - 1.28)	1.14	(1.05 - 1.23)	1.16	(1.04 - 1.29)
**6**	1.30	(1.22 - 1.39)	1.24	(1.12 - 1.37)	1.19	(1.04 - 1.37)
**7**	1.36	(1.24 - 1.50)	1.31	(1.12 - 1.52)	1.20	(0.97 - 1.48)
**8**	1.73	(1.48 - 2.02)	1.38	(1.08 - 1.76)	1.72	(1.23 - 2.42)
**9**	1.51	(1.18 - 1.92)	1.70	(1.16 - 2.50)	2.19	(1.18 - 4.06)
**10**	3.83	(2.34 - 6.26)	1.63	(0.84 - 3.18)	2.47	(0.92 - 6.66)
**11**	1.94	(0.77 - 4.84)	1.51	(0.49 - 4.71)	0.46	(0.05 - 4.46)
**12+**	0.82	(0.12 - 5.72)	0.86	(0.61 - 1.21)	0.60	(0.10 - 3.64)
**Current smoker**	1.09	(1.05 - 1.14)	1.01	(0.93 - 1.10)	1.03	(0.90 - 1.18)
**Heavy alcohol use**	1.46	(1.40 - 1.52)	1.25	(1.16 - 1.35)	1.41	(1.29 - 1.54)
**Medication use**						
**Antidepressants**	1.21	(1.19 - 1.24)	1.26	(1.21 - 1.32)	1.33	(1.26 - 1.41)
**Benzodiazepines**	1.14	(1.11 - 1.18)	1.12	(1.06 - 1.18)	1.11	(1.03 - 1.19)
**Anti-hypertensives**	1.02	(1.00 - 1.04)	1.04	(1.01 - 1.07)	1.02	(0.98 - 1.06)
**Anti-diabetic drugs**	1.06	(1.03 - 1.09)	1.02	(0.97 - 1.07)	1.06	(0.99 - 1.12)
**Insulin**	1.34	(1.27 - 1.42)	1.31	(1.20 - 1.43)	1.46	(1.31 - 1.61)
**ACB-2 drugs**	1.49	(1.39 - 1.60)	1.43	(1.27 - 1.62)	1.55	(1.31 - 1.83)
**ACB-3 drugs**	1.11	(1.08 - 1.14)	1.04	(1.00 - 1.09)	1.09	(1.02 - 1.15)
**Systemic steroids**	1.25	(1.22 - 1.29)	1.20	(1.14 - 1.25)	1.32	(1.24 - 1.40)
**Osteoporosis**	1.55	(1.51 - 1.58)	1.52	(1.46 - 1.58)	1.57	(1.48 - 1.65)
**Prior fracture history**	1.99	(1.95 - 2.03)	1.82	(1.76 - 1.89)	2.03	(1.93 - 2.13)

*Race compared to White, IMD compared to 1 (most deprived), CCI compared to CCI=0

© 2023 Tsang JY et al. *JAMA Ophthalmology.*

eTable 5. Sensitivity analysis 2 (single eye disease subanalysis)

eTable 5.1: Sensitivity analysis 2 (Single eye disease cohorts – falls)

	Cataract		AMD		Glaucoma	
	HR	95% CI	HR	95% CI	HR	95% CI
**Case**	1.44	(1.43 - 1.46)	1.91	(1.78 - 2.05)	2.40	(2.28 - 2.54)
**Age**	1 (Omitted)		1 (Omitted)		1 (Omitted)	
**Gender**	1 (Omitted)		1 (Omitted)		1 (Omitted)	
**Race***						
**Other**	0.70	(0.66 - 0.75)	0.64	(0.51 - 0.79)	0.83	(0.68 - 1.00)
**Asian**	0.86	(0.82 - 0.89)	0.73	(0.59 - 0.89)	0.75	(0.65 - 0.88)
**Black**	0.59	(0.56 - 0.62)	0.59	(0.45 - 0.76)	0.67	(0.57 - 0.78)
**Unknown**	0.20	(0.20 - 0.21)	0.15	(0.13 - 0.16)	0.18	(0.16 - 0.20)
**IMD***						
**2**	0.94	(0.92 - 0.96)	0.93	(0.87 - 0.99)	0.93	(0.87 - 1.00)
**3**	0.89	(0.88 - 0.91)	0.91	(0.85 - 0.97)	0.80	(0.74 - 0.86)
**4**	0.88	(0.86 - 0.89)	0.86	(0.80 - 0.92)	0.78	(0.72 - 0.84)
**5 (least deprived)**	0.85	(0.83 - 0.86)	0.86	(0.80 - 0.92)	0.77	(0.71 - 0.83)
**Unknown**	0.95	(0.79 - 1.15)	0.42	(0.19 - 0.89)	1.02	(0.48 - 2.17)
**Previous eye disease**						
**Cataract**	1 (Omitted)		1 (Omitted)		1 (Omitted)	
**Glaucoma**	1 (Omitted)		1 (Omitted)		1 (Omitted)	
**AMD**	1 (Omitted)		1 (Omitted)		1 (Omitted)	
**CCI Score***						
**1**	1.41	(1.38 - 1.44)	1.25	(1.12 - 1.40)	1.11	(1.01 - 1.23)
**2**	1.29	(1.27 - 1.32)	1.14	(1.02 - 1.26)	1.18	(1.07 - 1.31)
**3**	1.37	(1.34 - 1.41)	1.12	(0.98 - 1.28)	1.09	(0.95 - 1.25)
**4**	1.39	(1.35 - 1.43)	1.42	(1.20 - 1.68)	1.19	(1.00 - 1.41)
**5**	1.51	(1.45 - 1.57)	1.29	(1.03 - 1.61)	1.54	(1.18 - 2.00)
**6**	1.50	(1.42 - 1.59)	1.04	(0.79 - 1.36)	1.66	(1.16 - 2.38)
**7**	1.65	(1.52 - 1.80)	1.21	(0.75 - 1.96)	1.21	(0.70 - 2.07)
**8**	1.90	(1.65 - 2.18)	1.71	(0.74 - 3.94)	2.54	(1.13 - 5.69)
**9**	1.52	(1.24 - 1.87)	1.42	(0.43 - 4.76)	1.42	(0.21 - 9.60)
**10**	1.35	(0.97 - 1.89)	0.14	(0.01 - 1.88)	0.00	(0.00 - .)
**11**	2.44	(1.34 - 4.44)	0.54	(0.03 - 9.72)	0.00	(0.00 - .)
**12**	3.78	(0.95 - 14.97)	0.41	(0.02 - 6.82)	0.00	(0.00 - .)
**13**	1.26	(0.31 - 5.07)	1.00	(0.00 - 0.00)	0.00	(0.00 - .)
**14**	0.81	(0.13 - 5.18)	1.00	(0.00 - 0.00)	0.00	(0.00 - 0.00)
**Current smoker**	1.07	(1.04 - 1.11)	1.05	(0.93 - 1.18)	0.96	(0.73 - 1.26)
**Heavy alcohol use**	1.50	(1.45 - 1.55)	1.30	(1.10 - 1.53)	1.57	(1.37 - 1.81)
**Medication use**						
**Antidepressants**	1.26	(1.24 - 1.29)	1.33	(1.18 - 1.50)	1.43	(1.28 - 1.59)
**Benzodiazepines**	1.23	(1.20 - 1.27)	1.26	(1.08 - 1.46)	1.05	(0.91 - 1.22)
**Anti-hypertensives**	1.30	(1.28 - 1.32)	1.13	(1.04 - 1.22)	0.93	(0.87 - 1.00)
**Anti-diabetic drugs**	1.20	(1.17 - 1.23)	1.27	(1.12 - 1.45)	1.14	(1.01 - 1.29)
**Insulin**	1.34	(1.29 - 1.39)	1.45	(1.12 - 1.87)	1.31	(1.04 - 1.64)
**ACB-2 drugs**	1.58	(1.48 - 1.69)	1.62	(1.12 - 2.33)	1.80	(1.35 - 2.41)
**ACB-3 drugs**	1.22	(1.19 - 1.24)	1.10	(0.98 - 1.25)	1.21	(1.09 - 1.36)
**Systemic steroids**	1.28	(1.25 - 1.31)	1.29	(1.13 - 1.48)	1.34	(1.17 - 1.52)

© 2023 Tsang JY et al. *JAMA Ophthalmology.*

**Osteoporosis**	1.51	(1.48 - 1.54)	1.37	(1.22 - 1.53)	1.28	(1.13 - 1.45)
**Prior fracture history**	1.84	(1.82 - 1.87)	1.86	(1.76 - 1.98)	2.14	(1.99 - 2.31)

*Race compared to White, IMD compared to 1 (most deprived), CCI compared to CCI=0

eTable 5.2 Sensitivity analysis 2 (Single eye disease cohorts – fractures)

	Cataract		AMD		Glaucoma	
	HR	95% CI	HR	95% CI	HR	95% CI
**Case**	1.36	(1.33 - 1.38)	1.72	(1.57 - 1.88)	2.17	(2.02 - 2.33)
**Age**	1 (Omitted)		1 (Omitted)		1 (Omitted)	
**Gender**	1 (Omitted)		1 (Omitted)		1 (Omitted)	
**Race***						
**Other**	0.70	(0.65 - 0.76)	0.61	(0.46 - 0.80)	0.74	(0.58 - 0.95)
**Asian**	0.73	(0.69 - 0.78)	0.77	(0.60 - 1.00)	0.56	(0.45 - 0.68)
**Black**	0.34	(0.32 - 0.37)	0.45	(0.31 - 0.65)	0.43	(0.34 - 0.54)
**Unknown**	0.19	(0.18 - 0.19)	0.14	(0.12 - 0.16)	0.15	(0.14 - 0.17)
**IMD***						
**2**	0.95	(0.93 - 0.97)	0.93	(0.86 - 1.00)	0.91	(0.83 - 1.00)
**3**	0.90	(0.88 - 0.92)	0.93	(0.85 - 1.00)	0.80	(0.73 - 0.88)
**4**	0.88	(0.85 - 0.90)	0.89	(0.82 - 0.97)	0.76	(0.69 - 0.84)
**5 (least deprived)**	0.85	(0.83 - 0.87)	0.91	(0.83 - 0.99)	0.75	(0.68 - 0.83)
**Unknown**	1.06	(0.84 - 1.34)	0.43	(0.16 - 1.18)	1.14	(0.41 - 3.12)
**Previous eye disease Cataract**	1 (Omitted)		1 (Omitted)		1 (Omitted)	
**Glaucoma**	1 (Omitted)		1 (Omitted)		1 (Omitted)	
**AMD**	1 (Omitted)		1 (Omitted)		1 (Omitted)	
**CCI Score* 1**	1.21	(1.17 - 1.24)	1.17	(1.02 - 1.35)	1.02	(0.90 - 1.16)
**2**	1.17	(1.14 - 1.20)	1.08	(0.94 - 1.23)	1.07	(0.94 - 1.22)
**3**	1.21	(1.17 - 1.26)	1.03	(0.87 - 1.22)	0.94	(0.78 - 1.12)
**4**	1.24	(1.19 - 1.29)	1.39	(1.11 - 1.73)	1.06	(0.84 - 1.33)
**5**	1.28	(1.21 - 1.35)	1.13	(0.84 - 1.51)	1.36	(0.95 - 1.96)
**6**	1.33	(1.23 - 1.44)	1.19	(0.83 - 1.70)	1.30	(0.79 - 2.14)
**7**	1.45	(1.29 - 1.62)	0.93	(0.49 - 1.76)	1.20	(0.54 - 2.65)
**8**	1.71	(1.41 - 2.07)	1.47	(0.52 - 4.20)	1.96	(0.71 - 5.41)
**9**	1.32	(0.99 - 1.74)	0.85	(0.15 - 4.63)	1.72	(0.12 - 23.82)
**10**	1.70	(1.09 - 2.64)	0.00	(0.00 - .)	0.00	(0.00 - .)
**11**	2.01	(0.92 - 4.37)	0.00	(0.00 - .)	1.00	(0.00 - 0.00)
**12**	0.00	(0.00 - .)	0.00	(. - .)	1.00	(0.00 - 0.00)
**13**	0.82	(0.12 - 5.72)	1.00	(0.00 - 0.00)	0.00	(0.00 - .)
**14**	0.60	(0.10 - 3.64)	1.00	(0.00 - 0.00)	0.00	(0.00 - 0.00)
***Current smoker**	1.10	(1.05 - 1.15)	1.10	(0.95 - 1.27)	0.86	(0.61 - 1.21)
**Heavy alcohol use**	1.63	(1.56 - 1.70)	1.44	(1.16 - 1.78)	1.63	(1.36 - 1.95)
**Medication use Antidepressants**	1.24	(1.21 - 1.28)	1.32	(1.14 - 1.54)	1.40	(1.22 - 1.61)
**Benzodiazepines**	1.13	(1.09 - 1.18)	1.18	(0.98 - 1.42)	1.00	(0.83 - 1.21)
**Anti-hypertensives**	1.01	(0.99 - 1.03)	0.86	(0.78 - 0.96)	0.80	(0.73 - 0.88)
**Anti-diabetic drugs**	1.06	(1.03 - 1.10)	1.11	(0.94 - 1.31)	0.97	(0.82 - 1.15)
**Insulin**	1.48	(1.40 - 1.56)	1.83	(1.32 - 2.53)	1.90	(1.33 - 2.74)
**ACB-2 drugs**	1.52	(1.40 - 1.65)	1.69	(1.06 - 2.69)	1.07	(0.93 - 1.24)

© 2023 Tsang JY et al. *JAMA Ophthalmology.*

**ACB-3 drugs**	1.10	(1.07 - 1.14)	0.96	(0.82 - 1.12)	1.43	(1.06 - 1.93)
**Systemic steroids**	1.31	(1.27 - 1.35)	1.23	(1.04 - 1.46)	1.47	(1.25 - 1.74)
**Osteoporosis**	1.48	(1.44 - 1.52)	1.53	(1.34 - 1.76)	1.31	(1.12 - 1.53)
**Prior fracture history**	2.03	(1.98 - 2.07)	1.89	(1.76 - 2.04)	2.45	(2.22 - 2.71)

*Race compared to White, IMD compared to 1 (most deprived), CCI compared to CCI=0

© 2023 Tsang JY et al. *JAMA Ophthalmology.*

eTable 6. Multivariate-adjusted hazard ratios for incident fractures by body site with covariates

eTable 6.1 Hip

	Cataract		AMD		Glaucoma	
	HR	95% CI	HR	95% CI	HR	95% CI
**Case**	1.06	(1.04 - 1.09)	1.08	(1.03 - 1.13)	1.00	(0.94 - 1.07)
**Age**	1 (Omitted)		1 (Omitted)		1 (Omitted)	
**Gender**	1 (Omitted)		1 (Omitted)		1 (Omitted)	
**Race***						
**Other**	0.81	(0.72 - 0.92)	0.48	(0.36 - 0.65)	0.56	(0.41 - 0.75)
**Asian**	0.54	(0.48 - 0.61)	0.88	(0.71 - 1.09)	0.59	(0.47 - 0.74)
**Black**	0.19	(0.16 - 0.23)	0.27	(0.19 - 0.39)	0.25	(0.18 - 0.35)
**Unknown**	0.21	(0.20 - 0.23)	0.21	(0.18 - 0.24)	0.19	(0.17 - 0.22)
**IMD***						
**2**	0.91	(0.88 - 0.94)	0.89	(0.83 - 0.95)	0.91	(0.83 - 0.99)
**3**	0.86	(0.83 - 0.89)	0.89	(0.83 - 0.95)	0.93	(0.85 - 1.01)
**4**	0.82	(0.79 - 0.85)	0.84	(0.78 - 0.90)	0.81	(0.74 - 0.89)
**5 (least deprived)**	0.78	(0.75 - 0.81)	0.85	(0.79 - 0.91)	0.79	(0.72 - 0.86)
**Unknown**	1.05	(0.76 - 1.45)	0.82	(0.43 - 1.57)	1.04	(0.47 - 2.30)
**Previous eye disease**						
**Cataract**	1.00	(0.00 - 0.00)	0.87	(0.83 - 0.91)	0.90	(0.85 - 0.95)
**Glaucoma**	0.95	(0.92 - 0.98)	1.03	(0.97 - 1.09)	1.00	(0.00 - 0.00)
**AMD**	0.90	(0.87 - 0.93)	1.00	(0.00 - 0.00)	0.92	(0.86 - 0.99)
**CCI Score***						
**1**	1.04	(1.00 - 1.09)	0.99	(0.92 - 1.07)	0.00	(0.00 - 0.00)
**2**	0.95	(0.92 - 0.99)	0.91	(0.85 - 0.98)	1.08	(0.98 - 1.18)
**3**	1.06	(1.01 - 1.11)	1.10	(1.02 - 1.20)	0.96	(0.88 - 1.05)
**4**	1.13	(1.07 - 1.19)	1.14	(1.03 - 1.26)	1.05	(0.94 - 1.17)
**5**	1.11	(1.03 - 1.20)	1.19	(1.05 - 1.36)	1.17	(1.03 - 1.34)
**6**	1.08	(0.97 - 1.21)	1.12	(0.94 - 1.34)	1.09	(0.92 - 1.29)
**7**	1.59	(1.36 - 1.87)	1.37	(1.05 - 1.78)	1.21	(0.96 - 1.52)
**8**	1.60	(1.24 - 2.05)	1.30	(0.85 - 1.99)	0.78	(0.52 - 1.16)
**9**	1.35	(0.95 - 1.93)	1.14	(0.55 - 2.39)	1.81	(1.09 - 3.02)
**10**	1.33	(0.62 - 2.84)	1.03	(0.22 - 4.74)	2.65	(1.09 - 6.45)
**11**	4.28	(1.58 - 11.60)	0.62	(0.07 - 5.71)	1.69	(0.37 - 7.61)
**12+**	1.66	(0.22 - 12.48)	0.00	(0.00 - .)	4.44	(0.27 - 73.04)
**Current smoker**	1.14	(1.09 - 1.20)	1.03	(0.93 - 1.14)	1.00	(0.00 - 0.00)
**Heavy alcohol use**	1.14	(1.06 - 1.23)	0.95	(0.82 - 1.11)	1.00	(0.00 - 0.00)
**Medication use**						
**Antidepressants**	1.12	(1.08 - 1.16)	1.16	(1.07 - 1.26)	1.04	(0.87 - 1.23)
**Benzodiazepines**	1.06	(1.00 - 1.12)	1.06	(0.97 - 1.17)	1.21	(1.10 - 1.34)
**Anti-hypertensives**	0.78	(0.76 - 0.80)	0.84	(0.80 - 0.89)	1.05	(0.93 - 1.19)
**Anti-diabetic drugs**	1.07	(1.02 - 1.13)	1.07	(0.98 - 1.16)	0.81	(0.76 - 0.87)
**Insulin**	1.54	(1.41 - 1.68)	1.41	(1.20 - 1.64)	1.15	(1.04 - 1.29)
**ACB-2 drugs**	1.36	(1.20 - 1.54)	1.22	(0.97 - 1.52)	1.52	(1.26 - 1.83)
**ACB-3 drugs**	0.97	(0.93 - 1.02)	0.94	(0.87 - 1.02)	1.09	(0.80 - 1.49)
**Systemic steroids**	1.15	(1.09 - 1.20)	1.05	(0.97 - 1.15)	0.98	(0.89 - 1.08)
**Osteoporosis**	1.05	(1.01 - 1.10)	1.08	(1.01 - 1.16)	1.23	(1.11 - 1.37)
**Prior fracture history**	1.69	(1.64 - 1.75)	1.55	(1.46 - 1.64)	1.08	(0.99 - 1.19)

*Race compared to White, IMD compared to 1 (most deprived), CCI compared to CCI=0

© 2023 Tsang JY et al. *JAMA Ophthalmology.*

eTable 6.2 Spine

	Cataract		AMD		Glaucoma	
	HR	95% CI	HR	95% CI	HR	95% CI
**Case**	1.39	(1.34 - 1.44)	1.26	(1.18 - 1.35)	1.25	(1.14 - 1.37)
**Age**	1 (Omitted)		1 (Omitted)		1 (Omitted)	
**Gender**	1 (Omitted)		1 (Omitted)		1 (Omitted)	
**Race***						
**Other**	0.76	(0.62 - 0.95)	0.73	(0.44 - 1.22)	0.83	(0.52 - 1.33)
**Asian**	0.96	(0.83 - 1.11)	1.13	(0.83 - 1.54)	0.77	(0.57 - 1.04)
**Black**	0.29	(0.23 - 0.36)	0.33	(0.19 - 0.56)	0.29	(0.19 - 0.45)
**Unknown**	0.25	(0.23 - 0.28)	0.28	(0.22 - 0.35)	0.26	(0.21 - 0.33)
**IMD***						
**2**	1.07	(1.01 - 1.14)	1.15	(1.02 - 1.30)	1.11	(0.96 - 1.27)
**3**	1.08	(1.01 - 1.15)	1.13	(0.99 - 1.28)	1.10	(0.95 - 1.28)
**4**	1.02	(0.95 - 1.09)	1.07	(0.94 - 1.21)	0.98	(0.84 - 1.14)
**5 (least deprived)**	1.04	(0.98 - 1.12)	1.15	(1.01 - 1.32)	1.03	(0.88 - 1.21)
**Unknown**	0.84	(0.46 - 1.51)	0.82	(0.20 - 3.32)	0.59	(0.13 - 2.75)
**Previous eye disease**						
**Cataract**	1.00	(0.00 - 0.00)	1.41	(1.30 - 1.53)	1.39	(1.27 - 1.52)
**Glaucoma**	1.08	(1.02 - 1.14)	1.06	(0.97 - 1.17)	1.00	(0.00 - 0.00)
**AMD**	1.14	(1.09 - 1.20)	1.00	(0.00 - 0.00)	1.09	(0.97 - 1.22)
**CCI Score***						
**1**	1.55	(1.46 - 1.64)	1.54	(1.38 - 1.72)	1.43	(1.25 - 1.63)
**2**	1.35	(1.27 - 1.42)	1.19	(1.07 - 1.32)	1.08	(0.95 - 1.24)
**3**	1.36	(1.27 - 1.46)	1.23	(1.08 - 1.40)	1.27	(1.08 - 1.50)
**4**	1.28	(1.17 - 1.41)	1.22	(1.05 - 1.42)	1.34	(1.11 - 1.63)
**5**	1.25	(1.10 - 1.41)	1.11	(0.90 - 1.36)	1.45	(1.13 - 1.87)
**6**	1.36	(1.15 - 1.60)	1.39	(1.06 - 1.82)	1.26	(0.88 - 1.79)
**7**	1.59	(1.25 - 2.03)	1.24	(0.81 - 1.89)	1.59	(0.96 - 2.63)
**8**	1.97	(1.34 - 2.88)	1.73	(0.86 - 3.50)	1.61	(0.69 - 3.73)
**9**	2.57	(1.67 - 3.94)	2.51	(0.98 - 6.43)	1.97	(0.77 - 5.09)
**10**	0.90	(0.28 - 2.90)	1.66	(0.18 - 15.20)	2.98	(0.34 - 25.92)
**11**	7.28	(2.18 - 24.38)	2.69	(0.34 - 21.53)	0.95	(0.10 - 8.89)
**12+**	6.55	(0.66 - 65.01)	4.89	(0.30 - 78.55)	1.00	(0.00 - 0.00)
**Current smoker**	0.97	(0.87 - 1.09)	1.16	(0.92 - 1.46)	1.19	(0.86 - 1.66)
**Heavy alcohol use**	1.53	(1.39 - 1.68)	1.19	(0.99 - 1.44)	1.40	(1.13 - 1.73)
**Medication use**						
**Antidepressants**	1.18	(1.11 - 1.25)	1.27	(1.13 - 1.42)	1.24	(1.07 - 1.42)
**Benzodiazepines**	1.28	(1.18 - 1.39)	1.12	(0.97 - 1.30)	1.31	(1.10 - 1.57)
**Anti-hypertensives**	1.23	(1.18 - 1.29)	1.29	(1.19 - 1.40)	1.16	(1.05 - 1.28)
**Anti-diabetic drugs**	0.90	(0.83 - 0.96)	0.85	(0.75 - 0.97)	0.84	(0.72 - 0.99)
**Insulin**	1.02	(0.89 - 1.18)	1.10	(0.86 - 1.40)	1.18	(0.90 - 1.56)
**ACB-2 drugs**	1.56	(1.30 - 1.86)	1.40	(1.01 - 1.94)	1.68	(1.11 - 2.56)
**ACB-3 drugs**	1.26	(1.18 - 1.34)	1.20	(1.08 - 1.35)	1.19	(1.03 - 1.37)
**Systemic steroids**	1.65	(1.55 - 1.77)	1.41	(1.25 - 1.58)	1.63	(1.40 - 1.89)
**Osteoporosis**	2.75	(2.61 - 2.91)	2.75	(2.49 - 3.03)	2.66	(2.35 - 3.01)
**Prior fracture history**	1.89	(1.79 - 1.99)	1.70	(1.54 - 1.87)	2.09	(1.85 - 2.37)

*Race compared to White, IMD compared to 1 (most deprived), CCI compared to CCI=0

© 2023 Tsang JY et al. *JAMA Ophthalmology.*

eTable 6.3 Forearm

	Cataract		AMD		Glaucoma	
	HR	95% CI	HR	95% CI	HR	95% CI
**Case**	1.41	(1.38 - 1.43)	1.26	(1.21 - 1.32)	1.50	(1.43 - 1.58)
**Age**	1 (Omitted)		1 (Omitted)		1 (Omitted)	
**Gender**	1 (Omitted)		1 (Omitted)		1 (Omitted)	
**Race***						
**Other**	0.64	(0.57 - 0.73)	0.53	(0.39 - 0.72)	0.58	(0.44 - 0.76)
**Asian**	0.69	(0.64 - 0.76)	0.66	(0.54 - 0.80)	0.64	(0.55 - 0.76)
**Black**	0.32	(0.28 - 0.36)	0.33	(0.23 - 0.46)	0.36	(0.30 - 0.44)
**Unknown**	0.22	(0.21 - 0.23)	0.24	(0.22 - 0.28)	0.23	(0.21 - 0.26)
**IMD***						
**2**	0.98	(0.95 - 1.02)	0.99	(0.93 - 1.07)	0.98	(0.90 - 1.06)
**3**	0.93	(0.90 - 0.97)	0.98	(0.91 - 1.05)	0.89	(0.83 - 0.97)
**4**	0.94	(0.90 - 0.97)	0.98	(0.91 - 1.06)	0.86	(0.79 - 0.93)
**5 (least deprived)**	0.94	(0.91 - 0.98)	0.95	(0.88 - 1.03)	0.86	(0.79 - 0.93)
**Unknown**	1.03	(0.72 - 1.48)	1.06	(0.53 - 2.11)	0.66	(0.25 - 1.69)
**Previous eye disease Cataract**	1.00	(0.00 - 0.00)	1.35	(1.29 - 1.41)	1.45	(1.38 - 1.52)
**Glaucoma**	1.20	(1.16 - 1.23)	1.09	(1.03 - 1.15)	1.00	(0.00 - 0.00)
**AMD**	1.09	(1.05 - 1.12)	1.00	(0.00 - 0.00)	1.10	(1.02 - 1.17)
**CCI Score* 1**	1.33	(1.28 - 1.38)	1.20	(1.12 - 1.28)	1.22	(1.13 - 1.32)
**2**	1.23	(1.19 - 1.27)	1.15	(1.08 - 1.22)	1.07	(1.00 - 1.16)
**3**	1.29	(1.23 - 1.34)	1.13	(1.05 - 1.23)	1.13	(1.02 - 1.24)
**4**	1.22	(1.16 - 1.29)	1.11	(1.00 - 1.22)	1.04	(0.92 - 1.17)
**5**	1.32	(1.23 - 1.42)	1.09	(0.96 - 1.24)	1.02	(0.86 - 1.21)
**6**	1.29	(1.17 - 1.43)	1.17	(0.99 - 1.39)	1.00	(0.80 - 1.25)
**7**	1.36	(1.17 - 1.58)	1.45	(1.12 - 1.89)	1.34	(0.96 - 1.87)
**8**	1.66	(1.31 - 2.10)	1.59	(1.03 - 2.44)	1.96	(1.20 - 3.18)
**9**	0.85	(0.55 - 1.30)	1.55	(0.80 - 3.02)	1.34	(0.54 - 3.31)
**10**	2.26	(1.37 - 3.72)	1.33	(0.48 - 3.67)	1.56	(0.45 - 5.42)
**11**	0.94	(0.36 - 2.48)	1.06	(0.20 - 5.53)	2.19	(0.18 - 26.51)
**12+**	9.87	(1.02 - 95.56)	0.00	(0.00 - .)	0.00	(0.00 - .)
**Current smoker**	1.01	(0.94 - 1.08)	1.02	(0.89 - 1.18)	1.12	(0.91 - 1.39)
**Heavy alcohol use**	1.53	(1.45 - 1.62)	1.35	(1.20 - 1.52)	1.58	(1.39 - 1.79)
**Medication use Antidepressants**	1.29	(1.25 - 1.34)	1.25	(1.17 - 1.35)	1.36	(1.26 - 1.48)
**Benzodiazepines**	1.10	(1.05 - 1.15)	1.15	(1.06 - 1.26)	1.00	(0.90 - 1.11)
**Anti-hypertensives**	1.21	(1.18 - 1.24)	1.18	(1.12 - 1.24)	1.22	(1.15 - 1.29)
**Anti-diabetic drugs**	1.04	(1.00 - 1.09)	1.00	(0.92 - 1.08)	1.00	(0.92 - 1.10)
**Insulin**	1.25	(1.16 - 1.35)	1.31	(1.13 - 1.51)	1.40	(1.20 - 1.62)
**ACB-2 drugs**	1.35	(1.21 - 1.50)	1.55	(1.26 - 1.90)	1.66	(1.31 - 2.10)
**ACB-3 drugs**	1.06	(1.02 - 1.10)	0.99	(0.92 - 1.06)	1.05	(0.96 - 1.14)
**Systemic steroids**	1.13	(1.08 - 1.17)	1.20	(1.12 - 1.30)	1.12	(1.02 - 1.23)
**Osteoporosis**	1.38	(1.33 - 1.43)	1.31	(1.23 - 1.39)	1.39	(1.29 - 1.51)
**Prior fracture history**	1.85	(1.79 - 1.91)	1.72	(1.62 - 1.83)	1.90	(1.76 - 2.04)

*Race compared to White, IMD compared to 1 (most deprived), CCI compared to CCI=0

© 2023 Tsang JY et al. *JAMA Ophthalmology.*

eTable 6.4 Skull/facial bones

	Cataract		AMD		Glaucoma	
	HR	95% CI	HR	95% CI	HR	95% CI
**Case**	1.11	(1.03 - 1.19)	1.19	(1.03 - 1.37)	0.97	(0.82 - 1.15)
**Age**	1 (Omitted)		1 (Omitted)		1 (Omitted)	
**Gender**	1 (Omitted)		1 (Omitted)		1 (Omitted)	
**Race***						
**Other**	0.87	(0.65 - 1.16)	0.54	(0.26 - 1.14)	0.74	(0.42 - 1.33)
**Asian**	0.90	(0.73 - 1.11)	1.15	(0.71 - 1.86)	0.68	(0.44 - 1.04)
**Black**	0.50	(0.39 - 0.65)	0.52	(0.27 - 1.01)	0.59	(0.37 - 0.94)
**Unknown**	0.16	(0.14 - 0.19)	0.21	(0.14 - 0.32)	0.14	(0.09 - 0.20)
**IMD***						
**2**	0.93	(0.84 - 1.03)	0.81	(0.65 - 1.02)	0.92	(0.73 - 1.16)
**3**	0.90	(0.81 - 1.00)	0.98	(0.78 - 1.24)	0.87	(0.68 - 1.10)
**4**	0.94	(0.85 - 1.05)	0.89	(0.71 - 1.13)	0.80	(0.62 - 1.02)
**5 (least deprived)**	0.94	(0.84 - 1.05)	0.90	(0.70 - 1.15)	0.88	(0.68 - 1.13)
**Previous eye disease**						
**Cataract**	1.00	(0.00 - 0.00)	1.10	(0.96 - 1.27)	1.10	(0.94 - 1.29)
**Glaucoma**	1.05	(0.95 - 1.16)	1.12	(0.94 - 1.34)	1.00	(0.00 - 0.00)
**AMD**	0.94	(0.85 - 1.04)	1.00	(0.00 - 0.00)	0.90	(0.72 - 1.14)
**CCI Score***						
**1**	0.99	(0.88 - 1.11)	0.93	(0.74 - 1.18)	0.86	(0.65 - 1.14)
**2**	1.00	(0.89 - 1.11)	1.07	(0.87 - 1.32)	1.11	(0.87 - 1.43)
**3**	1.12	(0.97 - 1.28)	0.96	(0.74 - 1.24)	1.12	(0.81 - 1.53)
**4**	1.12	(0.95 - 1.32)	1.04	(0.77 - 1.42)	0.97	(0.67 - 1.42)
**5**	1.03	(0.82 - 1.30)	1.24	(0.84 - 1.84)	0.47	(0.25 - 0.88)
**6**	1.25	(0.93 - 1.69)	0.50	(0.26 - 0.96)	0.84	(0.37 - 1.88)
**7**	1.90	(1.27 - 2.84)	1.41	(0.63 - 3.15)	1.52	(0.59 - 3.89)
**8**	1.29	(0.69 - 2.40)	0.41	(0.09 - 1.89)	0.00	(0.00 - .)
**9**	0.82	(0.30 - 2.24)	2.19	(0.19 - 25.10)	4.22	(0.66 - 27.08)
**10**	0.00	(0.00 - .)	0.00	(. - .)	0.00	(0.00 - .)
**11**	0.00	(0.00 - .)	1.00	(0.00 - 0.00)	0.00	(0.00 - .)
**12+**	0.00	(0.00 - .)	1.00	(0.00 - 0.00)	0.00	(0.00 - .)
**Current smoker**	0.99	(0.83 - 1.17)	1.27	(0.84 - 1.92)	1.32	(0.75 - 2.31)
**Heavy alcohol use**	1.42	(1.19 - 1.68)	1.39	(0.97 - 1.99)	1.18	(0.81 - 1.72)
**Medication use**						
**Antidepressants**	1.17	(1.05 - 1.30)	1.63	(1.29 - 2.06)	1.41	(1.07 - 1.86)
**Benzodiazepines**	0.99	(0.84 - 1.16)	1.27	(0.96 - 1.67)	0.87	(0.60 - 1.27)
**Anti-hypertensives**	1.00	(0.92 - 1.08)	0.92	(0.78 - 1.09)	1.03	(0.86 - 1.25)
**Anti-diabetic drugs**	0.99	(0.87 - 1.13)	1.24	(0.97 - 1.59)	1.31	(0.99 - 1.73)
**Insulin**	1.13	(0.89 - 1.43)	0.72	(0.43 - 1.20)	1.29	(0.78 - 2.12)
**ACB-2 drugs**	1.80	(1.30 - 2.48)	1.34	(0.67 - 2.69)	5.25	(2.32 - 11.88)
**ACB-3 drugs**	1.20	(1.07 - 1.36)	0.92	(0.73 - 1.16)	1.13	(0.85 - 1.51)
**Systemic steroids**	1.02	(0.89 - 1.17)	0.86	(0.65 - 1.14)	1.31	(0.94 - 1.81)
**Osteoporosis**	1.18	(1.04 - 1.34)	1.34	(1.07 - 1.68)	1.21	(0.90 - 1.64)
**Prior fracture history**	1.38	(1.25 - 1.53)	1.33	(1.09 - 1.63)	1.75	(1.38 - 2.23)

*Race compared to White, IMD compared to 1 (most deprived), CCI compared to CCI=0

© 2023 Tsang JY et al. *JAMA Ophthalmology.*

eTable 6.5 Pelvis

	Cataract		AMD		Glaucoma	
	HR	95% CI	HR	95% CI	HR	95% CI
**Case**	1.10	(1.05 - 1.16)	1.13	(1.03 - 1.24)	1.06	(0.93 - 1.21)
**Age**	1 (Omitted)		1 (Omitted)		1 (Omitted)	
**Gender**	1 (Omitted)		1 (Omitted)		1 (Omitted)	
**Race***						
**Other**	0.80	(0.61 - 1.05)	0.80	(0.49 - 1.32)	0.93	(0.51 - 1.69)
**Asian**	0.91	(0.74 - 1.12)	0.91	(0.59 - 1.41)	0.93	(0.63 - 1.39)
**Black**	0.39	(0.29 - 0.53)	0.46	(0.23 - 0.90)	0.40	(0.22 - 0.71)
**Unknown**	0.18	(0.16 - 0.21)	0.15	(0.11 - 0.21)	0.21	(0.16 - 0.29)
**IMD***						
**2**	0.98	(0.91 - 1.06)	0.86	(0.75 - 0.99)	0.89	(0.74 - 1.07)
**3**	0.94	(0.87 - 1.02)	0.88	(0.77 - 1.02)	0.95	(0.79 - 1.14)
**4**	0.93	(0.86 - 1.00)	0.95	(0.82 - 1.10)	0.92	(0.76 - 1.11)
**5 (least deprived)**	0.99	(0.91 - 1.07)	0.92	(0.79 - 1.07)	1.06	(0.87 - 1.29)
**Unknown**	0.70	(0.28 - 1.74)	0.29	(0.04 - 2.42)	0.00	(0.00 - .)
**Previous eye disease**						
**Cataract**	1.00	(0.00 - 0.00)	0.99	(0.90 - 1.08)	0.99	(0.88 - 1.11)
**Glaucoma**	0.93	(0.87 - 1.00)	0.97	(0.87 - 1.09)	1.00	(0.00 - 0.00)
**AMD**	0.93	(0.87 - 0.99)	1.00	(0.00 - 0.00)	0.90	(0.78 - 1.04)
**CCI Score***						
**1**	0.99	(0.91 - 1.07)	1.04	(0.90 - 1.21)	1.16	(0.96 - 1.40)
**2**	1.05	(0.97 - 1.14)	0.98	(0.86 - 1.12)	1.22	(1.03 - 1.46)
**3**	1.15	(1.05 - 1.26)	1.02	(0.87 - 1.21)	1.06	(0.85 - 1.33)
**4**	1.30	(1.16 - 1.46)	1.11	(0.92 - 1.35)	0.99	(0.75 - 1.31)
**5**	1.26	(1.08 - 1.47)	1.11	(0.86 - 1.44)	1.42	(1.02 - 1.98)
**6**	1.40	(1.13 - 1.72)	1.49	(1.06 - 2.09)	1.55	(0.99 - 2.41)
**7**	0.88	(0.61 - 1.27)	1.24	(0.70 - 2.20)	1.53	(0.74 - 3.17)
**8**	0.89	(0.49 - 1.61)	1.74	(0.81 - 3.74)	2.24	(0.66 - 7.64)
**9**	1.07	(0.47 - 2.44)	1.08	(0.28 - 4.16)	5.32	(1.15 - 24.65)
**10**	3.86	(1.10 - 13.58)	4.49	(0.62 - 32.47)	4.41	(0.56 - 34.60)
**11**	4.35	(0.69 - 27.47)	0.00	(. - .)	10.82	(0.65 - 178.61)
**12+**	2.00	(0.12 - 32.49)	4.57	(0.27 - 76.68)	1.00	(0.00 - 0.00)
**Current smoker**	1.00	(0.90 - 1.12)	0.96	(0.78 - 1.18)	1.07	(0.76 - 1.51)
**Heavy alcohol use**	1.29	(1.11 - 1.50)	1.09	(0.80 - 1.48)	1.24	(0.88 - 1.76)
**Medication use**						
**Antidepressants**	1.09	(1.01 - 1.18)	1.10	(0.95 - 1.27)	1.17	(0.96 - 1.42)
**Benzodiazepines**	1.04	(0.93 - 1.15)	1.12	(0.94 - 1.34)	1.10	(0.86 - 1.39)
**Anti-hypertensives**	0.85	(0.80 - 0.90)	0.85	(0.76 - 0.94)	0.85	(0.75 - 0.98)
**Anti-diabetic drugs**	1.01	(0.91 - 1.12)	1.00	(0.84 - 1.19)	1.15	(0.92 - 1.44)
**Insulin**	1.72	(1.43 - 2.08)	1.13	(0.79 - 1.60)	1.17	(0.78 - 1.74)
**ACB-2 drugs**	1.27	(0.97 - 1.66)	1.84	(1.24 - 2.75)	1.18	(0.64 - 2.17)
**ACB-3 drugs**	1.00	(0.92 - 1.08)	0.93	(0.81 - 1.09)	0.90	(0.74 - 1.10)
**Systemic steroids**	1.38	(1.26 - 1.51)	1.17	(1.00 - 1.37)	1.38	(1.13 - 1.69)
**Osteoporosis**	1.68	(1.57 - 1.81)	1.79	(1.59 - 2.01)	2.00	(1.70 - 2.35)
**Prior fracture history**	1.83	(1.72 - 1.95)	1.70	(1.53 - 1.89)	2.05	(1.76 - 2.38)

*Race compared to White, IMD compared to 1 (most deprived), CCI compared to CCI=0

© 2023 Tsang JY et al. *JAMA Ophthalmology.*

eTable 6.6 Ribs/Sternum

	Cataract		AMD		Glaucoma	
	HR	95% CI	HR	95% CI	HR	95% CI
**Case**	1.18	(1.12 - 1.25)	1.10	(0.99 - 1.23)	1.26	(1.11 - 1.44)
**Age**	1 (Omitted)		1 (Omitted)		1 (Omitted)	
**Gender**	1 (Omitted)		1 (Omitted)		1 (Omitted)	
**Race***						
**Other**	0.62	(0.47 - 0.83)	0.70	(0.36 - 1.35)	0.86	(0.49 - 1.52)
**Asian**	0.72	(0.59 - 0.88)	1.08	(0.69 - 1.69)	0.58	(0.39 - 0.87)
**Black**	0.21	(0.15 - 0.29)	0.25	(0.12 - 0.53)	0.37	(0.22 - 0.61)
**Unknown**	0.17	(0.15 - 0.20)	0.24	(0.18 - 0.33)	0.21	(0.16 - 0.28)
**IMD***						
**2**	0.94	(0.87 - 1.02)	0.90	(0.76 - 1.07)	0.89	(0.74 - 1.06)
**3**	0.90	(0.83 - 0.98)	0.92	(0.77 - 1.09)	0.74	(0.61 - 0.89)
**4**	0.85	(0.78 - 0.93)	0.81	(0.68 - 0.97)	0.83	(0.69 - 1.00)
**5 (least deprived)**	0.78	(0.72 - 0.86)	0.80	(0.66 - 0.96)	0.74	(0.60 - 0.90)
**Unknown**	1.41	(0.74 - 2.68)	1.81	(0.59 - 5.56)	1.71	(0.44 - 6.66)
**Previous eye disease**						
**Cataract**	1.00	(0.00 - 0.00)	0.99	(0.88 - 1.10)	1.02	(0.90 - 1.15)
**Glaucoma**	0.96	(0.89 - 1.04)	0.89	(0.78 - 1.03)	1.00	(0.00 - 0.00)
**AMD**	0.86	(0.79 - 0.93)	1.00	(0.00 - 0.00)	0.99	(0.83 - 1.17)
**CCI Score***						
**1**	1.27	(1.16 - 1.38)	1.18	(0.99 - 1.41)	1.30	(1.06 - 1.58)
**2**	1.22	(1.12 - 1.33)	1.09	(0.92 - 1.28)	1.27	(1.05 - 1.53)
**3**	1.28	(1.16 - 1.42)	1.26	(1.04 - 1.52)	1.38	(1.09 - 1.74)
**4**	1.47	(1.29 - 1.67)	1.48	(1.18 - 1.85)	1.42	(1.08 - 1.87)
**5**	1.56	(1.33 - 1.83)	1.80	(1.36 - 2.39)	1.51	(1.05 - 2.19)
**6**	1.63	(1.31 - 2.02)	1.49	(1.03 - 2.17)	1.57	(0.99 - 2.51)
**7**	1.68	(1.22 - 2.33)	1.44	(0.79 - 2.64)	1.89	(0.91 - 3.91)
**8**	1.73	(0.95 - 3.15)	1.73	(0.70 - 4.26)	1.22	(0.36 - 4.08)
**9**	2.48	(1.19 - 5.14)	1.62	(0.39 - 6.67)	0.34	(0.03 - 3.39)
**10**	8.59	(3.21 - 22.96)	1.97	(0.12 - 32.27)	6.12	(1.05 - 35.78)
**11**	0.00	(0.00 - .)	1.00	(0.00 - 0.00)	0.00	(. - .)
**12+**	3.20	(0.18 - 56.65)	0.00	(0.00 - .)	1.00	(0.00 - 0.00)
**Current smoker**	1.12	(0.99 - 1.28)	0.96	(0.74 - 1.23)	0.69	(0.47 - 1.02)
**Heavy alcohol use**	1.59	(1.41 - 1.80)	1.41	(1.08 - 1.83)	1.77	(1.36 - 2.30)
**Medication use**						
**Antidepressants**	1.25	(1.15 - 1.35)	1.26	(1.05 - 1.50)	1.58	(1.29 - 1.93)
**Benzodiazepines**	1.14	(1.01 - 1.29)	1.17	(0.94 - 1.46)	1.06	(0.81 - 1.39)
**Anti-hypertensives**	0.94	(0.88 - 1.00)	1.00	(0.88 - 1.13)	0.90	(0.78 - 1.03)
**Anti-diabetic drugs**	0.98	(0.89 - 1.09)	0.97	(0.80 - 1.17)	1.00	(0.81 - 1.24)
**Insulin**	1.16	(0.97 - 1.39)	0.90	(0.63 - 1.30)	1.14	(0.80 - 1.63)
**ACB-2 drugs**	1.49	(1.15 - 1.93)	1.09	(0.67 - 1.78)	2.37	(1.38 - 4.06)
**ACB-3 drugs**	1.12	(1.03 - 1.23)	1.05	(0.88 - 1.26)	0.89	(0.72 - 1.09)
**Systemic steroids**	1.55	(1.42 - 1.71)	1.23	(1.03 - 1.47)	1.42	(1.16 - 1.74)
**Osteoporosis**	1.53	(1.39 - 1.67)	1.52	(1.29 - 1.78)	1.63	(1.32 - 2.01)
**Prior fracture history**	1.73	(1.60 - 1.87)	1.59	(1.38 - 1.83)	1.65	(1.38 - 1.97)

*Race compared to White, IMD compared to 1 (most deprived), CCI compared to CCI=0

© 2023 Tsang JY et al. *JAMA Ophthalmology.*

eTable 6.7 Lower leg

	Cataract		AMD		Glaucoma	
	HR	95% CI	HR	95% CI	HR	95% CI
**Case**	1.46	(1.41 - 1.51)	1.25	(1.17 - 1.34)	1.49	(1.38 - 1.61)
**Age**	1 (Omitted)		1 (Omitted)		1 (Omitted)	
**Gender**	1 (Omitted)		1 (Omitted)		1 (Omitted)	
**Race***						
**Other**	0.69	(0.59 - 0.82)	0.67	(0.43 - 1.04)	0.68	(0.48 - 0.94)
**Asian**	0.79	(0.71 - 0.88)	0.96	(0.73 - 1.27)	0.73	(0.58 - 0.91)
**Black**	0.54	(0.47 - 0.62)	0.51	(0.34 - 0.76)	0.52	(0.41 - 0.66)
**Unknown**	0.20	(0.19 - 0.22)	0.21	(0.17 - 0.26)	0.21	(0.18 - 0.24)
**IMD***						
**2**	0.94	(0.89 - 0.99)	0.97	(0.87 - 1.09)	0.99	(0.89 - 1.11)
**3**	0.87	(0.82 - 0.92)	0.87	(0.77 - 0.97)	0.87	(0.77 - 0.98)
**4**	0.91	(0.86 - 0.96)	0.94	(0.84 - 1.06)	0.88	(0.78 - 0.99)
**5 (least deprived)**	0.83	(0.78 - 0.88)	0.82	(0.72 - 0.93)	0.84	(0.74 - 0.96)
**Unknown**	0.75	(0.41 - 1.40)	0.43	(0.14 - 1.37)	0.40	(0.08 - 2.04)
**Previous eye disease**						
**Cataract**	1.00	(0.00 - 0.00)	1.03	(0.96 - 1.11)	1.19	(1.10 - 1.29)
**Glaucoma**	1.09	(1.04 - 1.14)	0.87	(0.79 - 0.96)	1.00	(0.00 - 0.00)
**AMD**	0.88	(0.84 - 0.93)	1.00	(0.00 - 0.00)	0.90	(0.81 - 1.01)
**CCI Score***						
**1**	1.23	(1.17 - 1.31)	1.27	(1.13 - 1.42)	1.27	(1.12 - 1.43)
**2**	1.19	(1.13 - 1.26)	1.13	(1.02 - 1.26)	1.13	(1.01 - 1.27)
**3**	1.30	(1.21 - 1.39)	1.23	(1.07 - 1.41)	1.22	(1.05 - 1.43)
**4**	1.32	(1.21 - 1.43)	1.37	(1.17 - 1.61)	1.40	(1.16 - 1.69)
**5**	1.50	(1.33 - 1.68)	1.14	(0.92 - 1.42)	1.31	(1.02 - 1.69)
**6**	1.72	(1.48 - 1.99)	1.74	(1.32 - 2.29)	1.64	(1.18 - 2.27)
**7**	1.54	(1.20 - 1.98)	1.14	(0.72 - 1.80)	1.27	(0.73 - 2.20)
**8**	1.97	(1.32 - 2.93)	0.94	(0.43 - 2.03)	2.86	(1.12 - 7.31)
**9**	2.10	(1.01 - 4.36)	2.21	(0.79 - 6.21)	2.42	(0.53 - 11.06)
**10**	2.59	(0.65 - 10.38)	0.95	(0.06 - 15.75)	0.00	(0.00 - .)
**11**	1.19	(0.28 - 5.11)	6.07	(0.53 - 69.05)	0.00	(0.00 - .)
**12**	0.26	(0.03 - 2.61)	0.00	(0.00 - .)	1.00	(0.00 - 0.00)
**Current smoker**	1.05	(0.94 - 1.16)	1.13	(0.89 - 1.43)	1.02	(0.72 - 1.45)
**Heavy alcohol use**	1.59	(1.47 - 1.74)	1.25	(1.03 - 1.53)	1.37	(1.14 - 1.64)
**Medication use**						
**Antidepressants**	1.24	(1.18 - 1.31)	1.30	(1.16 - 1.46)	1.45	(1.28 - 1.65)
**Benzodiazepines**	1.27	(1.18 - 1.37)	1.05	(0.90 - 1.22)	1.18	(1.00 - 1.39)
**Anti-hypertensives**	1.02	(0.98 - 1.06)	0.99	(0.91 - 1.08)	1.01	(0.92 - 1.10)
**Anti-diabetic drugs**	1.20	(1.12 - 1.27)	1.14	(1.00 - 1.29)	1.09	(0.95 - 1.25)
**Insulin**	1.95	(1.76 - 2.16)	2.11	(1.68 - 2.65)	2.25	(1.81 - 2.79)
**ACB-2 drugs**	1.74	(1.47 - 2.05)	1.80	(1.31 - 2.49)	1.43	(1.02 - 1.99)
**ACB-3 drugs**	1.10	(1.04 - 1.17)	1.14	(1.02 - 1.29)	1.09	(0.95 - 1.24)
**Systemic steroids**	1.29	(1.21 - 1.38)	1.29	(1.13 - 1.47)	1.39	(1.21 - 1.59)
**Osteoporosis**	1.26	(1.19 - 1.33)	1.30	(1.17 - 1.45)	1.40	(1.24 - 1.59)
**Prior fracture history**	2.38	(2.27 - 2.50)	2.26	(2.02 - 2.53)	2.43	(2.18 - 2.70)

*Race compared to White, IMD compared to 1 (most deprived), CCI compared to CCI=0

© 2023 Tsang JY et al. *JAMA Ophthalmology.*

eTable 7. Additional analysis (CPRD Aurum only analyses)

eTable 7.1: Additional analysis (CPRD Aurum only – falls)

	Cataract		AMD		Glaucoma	
	HR	95% CI	HR	95% CI	HR	95% CI
**Case**	1.37	(1.35 - 1.38)	1.24	(1.22 - 1.26)	1.38	(1.36 - 1.41)
**Age**	1 (Omitted)		1 (Omitted)		1 (Omitted)	
**Gender**	1 (Omitted)		1 (Omitted)		1 (Omitted)	
**Race***						
**Other**	0.72	(0.68 - 0.76)	0.68	(0.60 - 0.77)	0.68	(0.60 - 0.76)
**Asian**	0.85	(0.82 - 0.88)	0.83	(0.77 - 0.90)	0.79	(0.73 - 0.85)
**Black**	0.59	(0.57 - 0.61)	0.59	(0.53 - 0.65)	0.59	(0.55 - 0.64)
**Unknown**	0.23	(0.23 - 0.24)	0.27	(0.26 - 0.29)	0.25	(0.24 - 0.26)
**IMD***						
**2**	0.94	(0.92 - 0.95)	0.96	(0.93 - 0.99)	0.94	(0.91 - 0.97)
**3**	0.90	(0.89 - 0.92)	0.93	(0.90 - 0.96)	0.88	(0.85 - 0.91)
**4**	0.89	(0.87 - 0.90)	0.91	(0.88 - 0.94)	0.85	(0.82 - 0.89)
**5 (least deprived)**	0.86	(0.85 - 0.88)	0.88	(0.85 - 0.91)	0.83	(0.80 - 0.87)
**Unknown**	0.97	(0.83 - 1.14)	0.86	(0.63 - 1.16)	1.08	(0.74 - 1.58)
**Previous eye disease** **Cataract**	N/A	(0.00 - 0.00)	1.25	(1.22 - 1.27)	1.31	(1.28 - 1.34)
**Glaucoma**	1.17	(1.15 - 1.18)	1.12	(1.09 - 1.15)	N/A	(0.00 - 0.00)
**AMD**	1.07	(1.06 - 1.09)	N/A	(0.00 - 0.00)	1.05	(1.02 - 1.08)
**CCI Score*** **1**	1.42	(1.39 - 1.44)	1.31	(1.27 - 1.35)	1.35	(1.31 - 1.40)
**2**	1.28	(1.26 - 1.30)	1.19	(1.16 - 1.22)	1.20	(1.16 - 1.24)
**3**	1.38	(1.35 - 1.40)	1.30	(1.25 - 1.34)	1.33	(1.27 - 1.38)
**4**	1.37	(1.34 - 1.40)	1.30	(1.25 - 1.35)	1.31	(1.24 - 1.37)
**5**	1.47	(1.42 - 1.51)	1.33	(1.27 - 1.40)	1.33	(1.25 - 1.42)
**6**	1.48	(1.42 - 1.54)	1.44	(1.34 - 1.54)	1.55	(1.42 - 1.68)
**7**	1.61	(1.52 - 1.72)	1.66	(1.50 - 1.84)	1.43	(1.26 - 1.63)
**8**	1.70	(1.55 - 1.87)	1.64	(1.40 - 1.93)	2.10	(1.73 - 2.56)
**9**	1.83	(1.59 - 2.12)	1.74	(1.36 - 2.24)	2.43	(1.77 - 3.35)
**10**	1.87	(1.43 - 2.44)	1.94	(1.23 - 3.05)	2.08	(1.28 - 3.38)
**11**	2.59	(1.76 - 3.82)	0.93	(0.46 - 1.86)	1.56	(0.76 - 3.19)
**12+**	1.87	(1.17 - 3.01)	1.20	(0.53 - 2.70)	3.05	(1.14 - 8.17)
**Current smoker**	1.07	(1.04 - 1.10)	1.06	(1.00 - 1.11)	1.01	(0.93 - 1.09)
**Heavy alcohol use**	1.40	(1.36 - 1.44)	1.22	(1.16 - 1.29)	1.37	(1.30 - 1.45)
**Medication use** **Antidepressants**	1.26	(1.24 - 1.28)	1.32	(1.28 - 1.37)	1.39	(1.34 - 1.44)
**Benzodiazepines**	1.20	(1.18 - 1.23)	1.17	(1.12 - 1.21)	1.14	(1.08 - 1.20)
**Anti-hypertensives**	1.31	(1.29 - 1.32)	1.29	(1.27 - 1.32)	1.25	(1.22 - 1.28)
**Anti-diabetic drugs**	1.18	(1.16 - 1.20)	1.12	(1.09 - 1.16)	1.18	(1.14 - 1.23)
**Insulin**	1.33	(1.29 - 1.37)	1.25	(1.18 - 1.32)	1.36	(1.27 - 1.45)
**ACB-2 drugs**	1.56	(1.49 - 1.64)	1.46	(1.34 - 1.60)	1.74	(1.56 - 1.94)
**ACB-3 drugs**	1.20	(1.18 - 1.22)	1.12	(1.08 - 1.15)	1.14	(1.10 - 1.19)
**Systemic steroids**	1.26	(1.23 - 1.28)	1.17	(1.13 - 1.21)	1.25	(1.20 - 1.30)
**Osteoporosis**	1.52	(1.49 - 1.54)	1.47	(1.43 - 1.51)	1.48	(1.43 - 1.53)
**Prior fracture history**	1.79	(1.77 - 1.82)	1.75	(1.71 - 1.79)	1.83	(1.78 - 1.88)

*Race compared to White, IMD compared to 1 (most deprived), CCI compared to CCI=0, CCI compared to CCI 0

© 2023 Tsang JY et al. *JAMA Ophthalmology.*

eTable 7.2: Additional analysis (CPRD Aurum only – fractures)

	Cataract		AMD		Glaucoma	
	**HR**	**95% CI**	**HR**	**95% CI**	**HR**	**95% CI**
**Case**	1.29	(1.28 - 1.31)	1.18	(1.15 - 1.21)	1.28	(1.25 - 1.32)
**Age**	1 (Omitted)		1 (Omitted)		1 (Omitted)	
**Gender**	1 (Omitted)		1 (Omitted)		1 (Omitted)	
**Race***						
**Other**	0.71	(0.66 - 0.76)	0.61	(0.52 - 0.72)	0.62	(0.53 - 0.72)
**Asian**	0.75	(0.72 - 0.79)	0.89	(0.80 - 0.99)	0.67	(0.60 - 0.74)
**Black**	0.34	(0.32 - 0.36)	0.34	(0.28 - 0.40)	0.38	(0.33 - 0.42)
**Unknown**	0.21	(0.20 - 0.21)	0.22	(0.20 - 0.24)	0.22	(0.20 - 0.23)
**IMD***						
**2**	0.96	(0.94 - 0.98)	0.95	(0.91 - 0.98)	0.96	(0.91 - 1.00)
**3**	0.92	(0.90 - 0.93)	0.93	(0.89 - 0.97)	0.91	(0.87 - 0.95)
**4**	0.89	(0.87 - 0.91)	0.91	(0.88 - 0.95)	0.86	(0.82 - 0.90)
**5 (least deprived)**	0.88	(0.86 - 0.90)	0.90	(0.86 - 0.94)	0.85	(0.81 - 0.89)
**Unknown**	1.07	(0.88 - 1.31)	0.88	(0.60 - 1.30)	0.85	(0.51 - 1.40)
**Previous eye disease**						
**Cataract**	N/A	(0.00 - 0.00)	1.11	(1.08 - 1.14)	1.21	(1.17 - 1.25)
**Glaucoma**	1.06	(1.04 - 1.08)	1.01	(0.98 - 1.05)	N/A	(0.00 - 0.00)
**AMD**	0.99	(0.97 - 1.00)	N/A	(0.00 - 0.00)	1.00	(0.96 - 1.04)
**CCI Score***						
**1**	1.25	(1.22 - 1.28)	1.19	(1.14 - 1.24)	1.21	(1.15 - 1.27)
**2**	1.16	(1.13 - 1.18)	1.08	(1.04 - 1.12)	1.06	(1.01 - 1.11)
**3**	1.22	(1.19 - 1.25)	1.14	(1.09 - 1.20)	1.14	(1.07 - 1.20)
**4**	1.22	(1.18 - 1.26)	1.17	(1.10 - 1.23)	1.14	(1.06 - 1.22)
**5**	1.24	(1.19 - 1.29)	1.10	(1.02 - 1.18)	1.08	(0.98 - 1.18)
**6**	1.28	(1.21 - 1.35)	1.26	(1.15 - 1.39)	1.20	(1.06 - 1.36)
**7**	1.38	(1.27 - 1.51)	1.29	(1.11 - 1.49)	1.11	(0.92 - 1.35)
**8**	1.61	(1.41 - 1.84)	1.37	(1.09 - 1.73)	1.51	(1.13 - 2.02)
**9**	1.45	(1.18 - 1.77)	1.85	(1.27 - 2.67)	2.07	(1.31 - 3.26)
**10**	2.31	(1.63 - 3.26)	1.17	(0.58 - 2.39)	1.80	(0.89 - 3.64)
**11**	2.09	(1.23 - 3.56)	1.51	(0.64 - 3.61)	1.29	(0.47 - 3.54)
**12+**	1.18	(0.59 - 2.37)	0.65	(0.17 - 2.50)	0.39	(0.04 - 3.82)
**Current smoker**	1.08	(1.04 - 1.11)	1.04	(0.97 - 1.12)	1.03	(0.93 - 1.15)
**Heavy alcohol use**	1.48	(1.43 - 1.53)	1.24	(1.15 - 1.33)	1.40	(1.30 - 1.50)
**Medication use**						
**Antidepressants**	1.22	(1.19 - 1.24)	1.25	(1.20 - 1.30)	1.34	(1.28 - 1.41)
**Benzodiazepines**	1.14	(1.11 - 1.17)	1.12	(1.06 - 1.18)	1.08	(1.01 - 1.15)
**Anti-hypertensives**	1.02	(1.01 - 1.04)	1.03	(1.00 - 1.07)	1.03	(0.99 - 1.07)
**Anti-diabetic drugs**	1.07	(1.04 - 1.09)	1.02	(0.98 - 1.07)	1.07	(1.02 - 1.13)
**Insulin**	1.44	(1.38 - 1.50)	1.33	(1.23 - 1.45)	1.47	(1.34 - 1.61)
**ACB-2 drugs**	1.48	(1.39 - 1.58)	1.39	(1.24 - 1.57)	1.60	(1.38 - 1.85)
**ACB-3 drugs**	1.09	(1.07 - 1.12)	1.04	(1.00 - 1.08)	1.07	(1.02 - 1.12)
**Systemic steroids**	1.28	(1.25 - 1.31)	1.21	(1.16 - 1.27)	1.31	(1.24 - 1.39)
**Osteoporosis**	1.52	(1.49 - 1.55)	1.49	(1.44 - 1.55)	1.57	(1.50 - 1.65)
**Prior fracture history**	1.97	(1.94 - 2.01)	1.81	(1.75 - 1.87)	2.03	(1.95 - 2.12)

*Race compared to White, IMD compared to 1 (most deprived), CCI compared to CCI=0, CCI compared to CCI 0

© 2023 Tsang JY et al. *JAMA Ophthalmology.*
