# Supplementary material for: Risk of Falls and Fractures in Individuals With Cataract, Age-Related Macular Degeneration, or Glaucoma
Source: JAMA Ophthalmol. 2023 Dec 28;142(2):96–106. doi: 10.1001/jamaophthalmol.2023.5858 (PMC10870181; doi:10.1001/jamaophthalmol.2023.5858)
Supplement: Supplement 2. — Data sharing statement [file jamaophthalmol-e235858-s002.pdf]

Data Sharing Statement

Tsang. Risk of Falls and Fractures in Individuals With Cataract, Age-Related Macular Degeneration, or Glaucoma. *JAMA Ophthalmol*. Published December 28, 2023. doi:10.1001/jamaophthalmol.2023.5858

Data
Data available: No

Additional Information
Explanation for why data not available: In this study we used anonymized patient-level data from the CPRD that are not publicly available due to confidentiality considerations. However, researchers can access CPRD's databases by contacting the CPRD. Details of the application process and conditions of access are available at https://www.cprd.com/data-access.
